# Nanoantioxidant Materials: Nanoengineering Inspired by Nature

**DOI:** 10.3390/mi14020383

**Published:** 2023-02-04

**Authors:** Fotini Fragou, Annita Theofanous, Yiannis Deligiannakis, Maria Louloudi

**Affiliations:** 1 Laboratory of Biomimetic Catalysis & Hybrid Materials, Department of Chemistry, University of Ioannina, GR-45110 Ioannina, Greece; 2 Laboratory of Physical Chemistry of Materials & Environment, Department of Physics, University of Ioannina, GR-45110 Ioannina, Greece

**Keywords:** nanoantioxidants, reactive oxygen species (ROS), reactive nitrogen species (RNS), free radicals, nanoengineering, biomimetics, hybrid nanomaterials, surface functionalization, antioxidant nanostructures, advanced nanoantioxidant, hydrogen atom transfer (HAT)/proton-coupled electron transfer (PCET)

## Abstract

Oxidants are very active compounds that can cause damage to biological systems under specific environmental conditions. One effective way to counterbalance these adverse effects is the use of anti-oxidants. At low concentrations, an antioxidant is defined as a compound that can delay, control, or prevent an oxidative process. Antioxidants exist in plants, soil, and minerals; therefore, nature is a rich source of natural antioxidants, such as tocopherols and polyphenols. In nature, antioxidants perform *in tandem* with their bio-environment, which may tune their activity and protect them from degradation. In vitro use of antioxidants, i.e., out of their biomatrix, may encounter several drawbacks, such as auto-oxidation and polymerization. Artificial nanoantioxidants can be developed via surface modification of a nanoparticle with an antioxidant that can be either natural or synthetic, directly mimicking a natural antioxidant system. In this direction, state-of-the-art nanotechnology has been extensively incorporated to overcome inherent drawbacks encountered in vitro use of antioxidants, i.e., out of their biomatrix, and facilitate the production and use of antioxidants on a larger scale. Biomimetic nanoengineering has been adopted to optimize bio-medical antioxidant systems to improve stability, control release, enhance targeted administration, and overcome toxicity and biocompatibility issues. Focusing on biotechnological sciences, this review highlights the importance of nanoengineering in developing effective antioxidant structures and comparing the effectiveness of different nanoengineering methods. Additionally, this study gathers and clarifies the different antioxidant mechanisms reported in the literature and provides a clear picture of the existing evaluation methods, which can provide vital insights into bio-medical applications.

## 1. Introduction

Natural and synthetic antioxidants act as regulating agents, controlling or inhibiting the formation and damaging effect of free radical-moieties, thus preventing oxidation mechanisms towards oxidizable substrates, even at low concentrations [1,2]. Depending on the targeted application, e.g., food, cosmetics, or biomedical industry, etc., the definition of an antioxidant molecule might vary, incorporating the parameters defined by the specific scientific field [3]. Numerous studies in the last two decades confirm that the development of antioxidants is an increasingly emerging field. In this context, understanding the fundamental structure/function principles of natural antioxidants, and implementing this knowledge into nanotechnology, can drive the development of innovative biomimetic nanoantioxidants. 

The importance of biomimetic nanoantioxidants in biomedical sciences is reflected in the critical redox-regulated alterations induced in a cell environment in the presence of free radicals and ROS. Vital biological functions, e.g., signaling, are mainly regulated by redox reactions [4]. An intriguing aspect to grasp is what happens under increased concentrations of free radicals and ROS, which may cause an imbalance between beneficial and unwanted moieties and, thus, severe toxicity to cells [5].

Among the reported specific biomedical applications, antioxidants have been utilized in bone-defect healing [4] and bone-regeneration materials such as bioactive glasses [5]. Depending on the antioxidant structure, the surrounding environmental conditions, and the specific targeted application, different interfacial processes lead to efficient control of the adverse effects of damaging chemical moieties. Nanodrug carriers, nano-based imaging, gene delivery, drug loading, and immunoassays reflect some state-of-the-art applications [6]. Moreover, the intracellular and extracellular release of antioxidants facilitates the control of oxidative stress and thus hinders pro-inflammatory phenomena [7]. Reversing aging mechanisms underpin much of the cosmetic and biomedical industry, while antioxidant mechanisms are also incorporated into developing pharmaceuticals and dietary supplements. Lastly, the capacity of natural antioxidants to counteract coronavirus effects has been recently investigated [8]. 

Several reviews have been published so far, covering different aspects of the nanoantioxidant field [1], [8,9,10,11,12,13,14]. For instance, Shah et al. describe nanoantioxidants as the fourth generation of antioxidants, but point out that new precise methods for measuring antioxidant activity are needed [1]. 

The present review aims to enhance the knowledge around specific issues correlated with nanoengineering aspects of novel antioxidant materials. More specifically, the focus of this review study is: (i) to gather and clarify the different nanoantioxidant mechanisms reported in the literature concerning nanoantioxidant materials, (ii) to provide a comprehensive picture of the existing evaluation methods of antioxidant activity in relation to their applicability in hybrid nanoantioxidants, (iii) to highlight key parameters in nanoengineering, towards the development of efficient antioxidant structures, via case-studies, and, finally, (iv) to compare the efficiency of different nanoengineering methods, in terms of optimization of the antioxidant activity by the nanohybrids. 

In this context, the review is divided into five main sections. In Section 2, a detailed presentation of antioxidants from natural sources is provided, the inherent properties of which inspire biomimetic artificial antioxidants nanoengineering. Moreover, an introduction to some characteristic artificial nanoantioxidant systems is presented. Section 3 presents the different oxidants that have to be counterbalanced by antioxidant systems, such as free radicals, ROS, and RNS, while emphasizing the different antioxidant mechanisms. Evaluation methods and utilized ways of expressing antioxidant activity are covered in Section 4. Finally, in Section 5, different nanoengineering concepts leading to advanced functional antioxidant structures are described. More specifically, in Section 5, four main concepts of nanoengineering are analyzed in detail, including artificial nanoantioxidants produced directly from biological sources, non-covalent and covalent surface modification processes, and nanozymes. 

## 2. Natural and Artificial Biomimetic Antioxidants 

A compound is generally defined as an antioxidant when—found in small concentrations—it can delay, control, or prevent an oxidative process. Depending on their action mechanism that defines their efficiency, antioxidants are classified as primary, secondary, and—with the recently introduced term—tertiary antioxidants [3]. Natural antioxidants are known to neutralize radicals by following one of the two fundamental reaction pathways, i.e., either hydrogen atom transfer [15] or sequential electron–proton transfer [16] mechanisms, and a minimal amount are needed to scavenge many radicals [1]. On the other hand, secondary antioxidants [1] need a larger amount to act as radical scavengers; in this case, the antioxidant concentration must be equal to the concentration of free radicals [3]. Hereafter, a detailed presentation of antioxidants from natural sources is provided, whose inherent properties can inspire the engineering of biomimetic artificial nanoantioxidants. Additionally, an introduction to some characteristic artificial nanoantioxidant systems is presented here, but will be fully-analyzed in detail in Section 5. 

### 2.1. Natural Antioxidants

Antioxidant systems in nature extend into many categories, with their primary distinction being enzymatic and non-enzymatic [17]. Enzymatic antioxidants are well-studied systems that primarily include glutathione peroxidase (GPx), catalase (CAT), and superoxide dismutase (SOD), known to protect the targeted cells from free radical damage [18]. Non-enzymatic antioxidant systems include polyphenols, carotenoids, vitamins, minerals, and other antioxidants. Other antioxidants include proteins, such as albumin and ceruloplasmin, and non-proteins, such as uric acid, bilirubin, etc. [17]. 

Phenolic/polyphenolic compounds

The largest category of antioxidants includes polyphenols. Found in many plants, vast literature and great interest in these antioxidants have arisen, and their products have gained much attention, as they are associated with many health benefits [19]. Phenolic compounds include dyes and flavorings, ranging from simple to conjugated complexes [20]. They bear one (or more) aromatic rings with one (or more) hydroxyl moieties [21]. The antioxidant activity of polyphenols is primarily due to hydroxyl groups located on the benzene rings [22]. In the work of Platzer et al. [21] the structure–activity relationship of phenolic compounds is described. When they bear more than two hydroxyl groups, the term polyhydroxy phenolic compound is used, while compounds bearing more than one phenol moiety are called polyphenolic [3]. Polyphenolic acids also belong to the category of polyphenols. 

One of the most discussed and investigated phenolic acids is gallic acid (GA) (3,4,5-trihydroxy benzoic acid). Due to its unique properties, GA, which can be found in plants, is involved in the medical and food industries [23]. As a low molecular weight compound, GA is considered one of the best natural antioxidants, with a remarkable ability to neutralize radicals, prevent lipid peroxidation, and chelate metal ions. GA acquires all these properties and antioxidant activity due to its three hydroxyl groups [24]. Another well-known natural antioxidant is caffeic acid (CA) (3,4-dihydroxycinnamic acid) which presents cardiac, immunomodulatory, and anti-inflammatory activities. CA is a natural phenolic acid found in plants and some herbs at high levels, as well as in wine, and it is considered one of the essential natural phenols in argan oil [25]. Rosmarinic acid (RA) can be produced by extracting *Rosemarinus Officinalis*L. [26]. It is a dimer of caffeic acid, and it consists of caffeic acid and (R)-(+)-3-(3, 4-ihydroxy phenyl) lactic acid [20]. It contains two hydroxyl groups on different phenolic rings; both positioned at the o-position. As an antioxidant, it can inhibit the action of xanthine oxidase [26]. Similarly, tannic acid is a natural hydrolysable polyphenol composed of ten gallic acid molecules, and it has many applications in pharmaceuticals and cosmetics. It can also interact with enzymes and inhibit their action in the body [27]. A natural hydroxyanthraquinone pigment used in the pharmaceutical industry is carminic acid, which can neutralize free radicals and ROS [28]. Another distinct category is humic acids, namely natural antioxidants composed of polyphenolic compounds, carboxylic acids, carbonyls, and quinoids that can scavenge free radicals [29]. Finally, curcumin (1,7-bis(4-hydroxy-3-methoxyphenyl)-1,6-heptadiene-3,5-dione) is a hydrophobic polyphenol derived from the herb *Curcuma Longa* frequently used as an antioxidant in China and India [30]. The *Pleurotus Florida* mushroom, besides containing several nutrients, such as dietary fiber, minerals, etc., also exhibits antioxidant activity arising from its methanol extract, 3-methoxy-4-hydroxy cinnamic acid, also known worldwide as ferulic acid [31]. 

Flavonoids 

In vegetables, a compound called *morin* (2′,3,4′,5,7-pentahydroxyflavone) belongs to phenolic compounds. Morin can have a protective effect against cardiovascular diseases [32]. Quercetin is a unique natural flavonol compound and contains five hydroxyl groups and one carbonyl group. It can form complexes with many metal ions, and it presents good antioxidant activity [33]. Quercetin (2-(3, 4-dihydroxyphenyl)-3, 5,7-trihydroxy-4H-1-benzopyran-4-one) can be used in clinical applications [34], while rutin (quercetin-3-O-[α-L-rhamnosyl-(1→6)-β-D- glucopyranoside]) is a natural flavonoid found in fruits, such as oranges and lemons [35]. 3-Hydroxy-4′-methoxyflavone exhibits, finally, high antioxidant activity, since it contains an electron-rich substituent [36].

Vitamins 

Trolox, although not a vitamin, is an extensively used analogue to alpha-tocopherol, except that a more hydrophilic carboxyl group replaces one side chain. Trolox is used more than a-tocopherol because of its solubility in water, and because it presents higher antioxidant activity [37]. Vitamin E, or α-tocopherol, is said to be the most potent natural lipophilic antioxidant, enabling it to fight lipid peroxidation in cells through chain-breaking reactions [37,38]. 

Polysaccharides

Hyaluronic acid (Hya) is a natural polysaccharide consisting of D-Glucuronic acid and N-Acetyl-D-Glucosamine. Hya is widely used in cosmetics, and is known for its antioxidant activity [15]. It is a ubiquitous compound, due to its wide distribution in vertebrates and its presence as a component of the cell envelope of many bacterial strains [39]. Similarly, gum arabic is a natural polysaccharide that can interact with hydrophobic drugs through its hydrophobic internal structure [40]. 

Polymers and Carotenoids

Chitosan is a cationic polymer used for targeted drug delivery [41]. It can be obtained from both marine and animal sources. It can also be extracted from mushrooms and other fungi [42]. It has gained significant importance due to its unique characteristics [43] and biological activity [42]. Similarly, carotenoids are derived from fruits and vegetables (e.g., potatoes and carrots) and are considered phytochemical antioxidants [17]. Carbon-based materials exhibit significant hydroxyl radical scavenging activity and can also be produced from physical sources [44,45]. Melanin’s antioxidant and photoprotective properties have been studied in this context [46,47].

Minerals

Complementary to phenolic antioxidants, vitamins, and carotenoids, minerals can exhibit specific antioxidant activity. Nutrients present in the human body, such as selenium (Se), provide many advantages, and have been proven effective in cancer treatment [37]. Among metals, gold nanoparticles (Au NPs) exhibit notable antioxidant activity [38]. Carbon-based materials exhibit antioxidant properties owing to their structural features, and sp^2^-hybridized carbon atoms that act as “traps” of free radicals [48]. Among them, shungite is known to exhibit specific antioxidant activity [49,50].

Engineered cerium oxide nanoparticles, known as nanoceria, hold a critical position among the biomimetic nanoantioxidants, primarily due to their unique physicochemical characteristics. Nanoceria, a rare earth oxide, is known to capture, store and release oxygen from its surface [51]. The cerium atom in nanoceria possesses two stable oxidation states, Ce^3+^ and Ce^4+^, unlike other lanthanides. Therefore, CeONPs possess self-regenerating properties, namely an internal continuous redox-regulated conversion of Ce^3+^/Ce^4+^. This auto-reduction mechanism emerges as an enzyme-mimicking activity that determines the antioxidant, antiradical, antibacterial, and anticancer activity of CeONPs; it is called oxygen storage capacity (OCS), and it enables oxidation/reduction reactions central to many applications [51]. CeONPs can clean up ROS and RNS due to their enzyme-mimicking activity [52]. More specifically, CeONPs can utilize this inherent redox-regeneration capacity to scavenge free radicals formed in healthy cells, due to various intrinsic or extrinsic factors, leading to cell death. Mimicking the catalytic activity of the enzyme superoxide dismutase (SOD), superoxide radicals (O_2_^™●^) can be neutralized through their reduction to H_2_O_2_. The H_2_O_2_ is, accordingly, catalytically converted to water, adopting the catalase CAT-mimetic activity. This way, the physiological cell function can be restored in the presence of nanoceria. Except for cerium oxide, yttrium oxide (or yttria), iron oxide, and manganous phosphate exhibit SOD- and CAT-mimetic activity by catalyzing disproportionation reactions of the superoxide radical and hydrogen peroxide [53].

### 2.2. Artificial Biomimetic Nanoantioxidants

The unique chemistry of natural antioxidants has inspired the creation of artificial biomimetic systems. This technological approach aims mainly to optimize efficacy or decrease the inherent structural drawbacks of natural antioxidants. Nanotechnology has given rise to a new wave of antioxidants that can be utilized as disease-preventive or therapeutic agents against redox-regulated cell malfunctions [18]. Concurrently, drawbacks of natural antioxidants, such as sensitivity to environmental factors, light, or pH, can be eliminated [19]. These artificial nanoantioxidants mimic natural antioxidant mechanisms in radical scavenging, an activity provided by the characteristic functional moieties they bear on their surface. Nanoengineering provides numerous alternative routes for the fabrication of advanced structures, which can be incorporated into medicine and biotechnology to prevent or treat cancer, aging-related, or neurodegenerative diseases [1]. Finally, modifying well-studied mechanisms provided by nature ensures the efficacy of the engineered materials.

**Figure 1:**
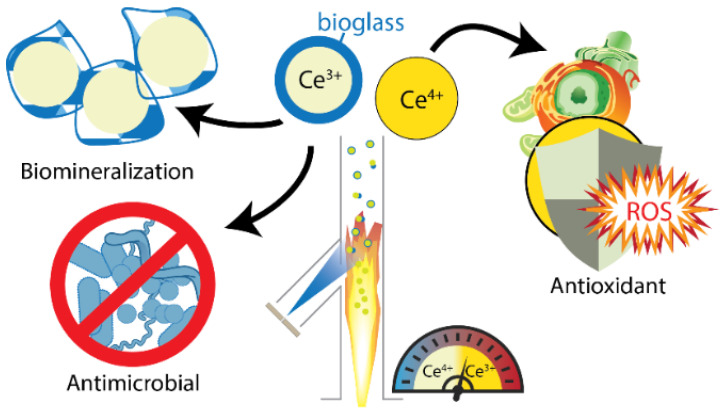
Engineered nanoceria structures are used as stand-alone materials, as support matrices for nanohybrids, or as coatings. The activity of nanoceria, whether acting as an antioxidant, protecting mammalian cells from oxidative death, or suppressing microbial growth, is regulated by specific environmental conditions. Reprinted (adapted) with permission from [54]. Copyright 2023 American Chemical Society.

Biomimetic artificial antioxidant systems include nanoparticles with intrinsic antioxidant properties, as well as nanoparticles that bear an immobilized antioxidant moiety [53]. Nanoceria (CeO_2_) (see Figure 1), that exists equally as natural and artificial antioxidant nanostructures, presents intrinsic antioxidant properties due to the capacity of the cerium atom to convert between its two oxidation states (Ce^3+^/Ce^4+^) [54]. This enzyme-mimicking activity is extensively studied for its potential utilization in biomedical applications [54,55]. Engineered nanoceria structures are used as stand-alone materials, as support matrices for nanohybrids, or as coatings. The activity of nanoceria, whether acting as an antioxidant, protecting mammalian cells from oxidative death, or suppressing microbial growth, is regulated by specific environmental conditions. For instance, when penetrating a cell with low pH, the CAT-mimetic activity of CeONPs is inhibited, which leads to a pro-oxidant effect, namely an intracellular increase in ROS [56]. This phenomenon could be used in designing nanoceria-based anticancer drugs, since the acidic pH is a characteristic of cancer cells, unlike healthy cells, which operate at a neutral pH [56]. This way, the SOD-mimetic mechanism of CeONPs, together with the inhibition of the CAT-mimetic activity, increases the concentration of H_2_O_2_ and thus leads to oxidative damage to the cancer cell [56]. In recent years, a substantial investigation has been carried out to modulate nanoceria’s redox performance by changing the crystal nano environment [51]. Inducing different kinds of defects in the lattice alters the oxygen storage capacity (OSC). The presence of oxygen vacancies in the lattice facilitates the free radical scavenging capacity. It enhances the overall antioxidant, antiradical, or antibacterial activity, which is of significant interest, among others, in biomedical applications [51]. Medicine has traditionally used molecule-based therapeutic agents, and the active molecule’s efficacy was adjusted by immobilizing functional moieties. Conversely, nanoceria is a crystal, and its efficacy, as the active ingredient, can be tuned by defect engineering for the controlled release of oxygen [51].

Metallic NPs, such as Au, Ag, Pt, and Zn and their oxides, are produced in large amounts per year utilizing wet-chemistry and flame pyrolysis methods [57]. Zinc oxides are extensively produced to be applied in biomedicine, due to their antioxidant combined with anti-inflammatory and antibacterial properties. Engineered iron nanoparticles are equally crucial for obtaining magnetic nanoantioxidants, which an external magnetic field can control [58,59]. The self-assembling properties of engineered gold NPs enable their use as supports and coating materials for increased biocompatibility [38]. Similarly, carbon-based nanomaterials, such as nanotubes or fullerenes, present antioxidant activity [44,60]. Finally, nanosilica is a typical inert matrix incorporated into biomimetic nanoantioxidant structures, either as a support matrix or as a coating, to enhance stability, biocompatibility, and low toxicity [61].

## 3. Oxidant Species and Counterbalancing Antioxidant Mechanisms

Oxidation reactions, incorporating different oxidant species, occupy an important place among the biochemical processes of the cellular metabolism of living organisms. However, their role can be reversed to being toxic under specific conditions that lead to their increased concentration. Autoxidation is also a ubiquitous organic reaction accompanying substrates with C-H bonds in an oxygen-rich environment. Molecular O_2_ can be spontaneously inserted into a substrate, i.e., via radical chain-reactions. The billions spent annually on antioxidant technologies reflect the enormous economic impact of these reactions [62].

**Figure 2:**
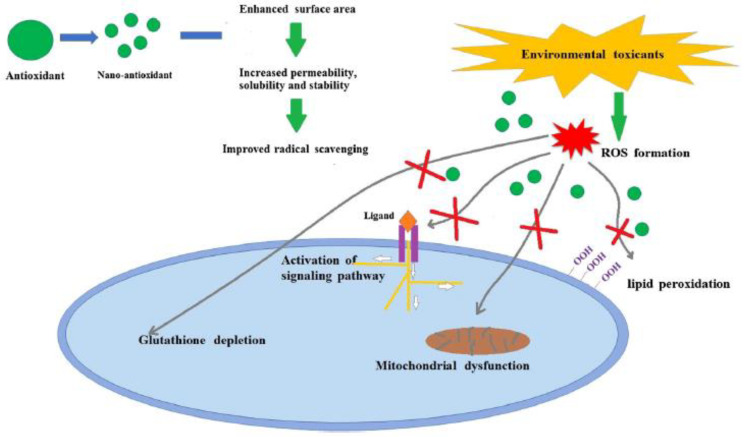
Schematic depiction of oxidative-stress related damage. Nanoantioxidants counterbalance these adverse effects due to their advanced structure. Reprinted (adapted) with permission from [63] Elsevier.

To prevent adverse effects, for instance, oxidative or nitrosative stress, induced by the overproduction of oxidants, cells utilize antioxidants as defense mechanisms [12]. Additionally to biological systems, Figure 2 depicts the oxidative-stress related damage into the eco-system, where nanoantioxidants counterbalance these adverse effects due to their advanced structure [63]. Thus, antioxidant species have numerous moieties to counterbalance, such as free radicals, reactive oxygen species (ROS), reactive nitrogen species (RNS), and other oxidants [13].

### 3.1. Free Radicals, ROS, RNS, and Other Oxidant Species

**Figure 3:**
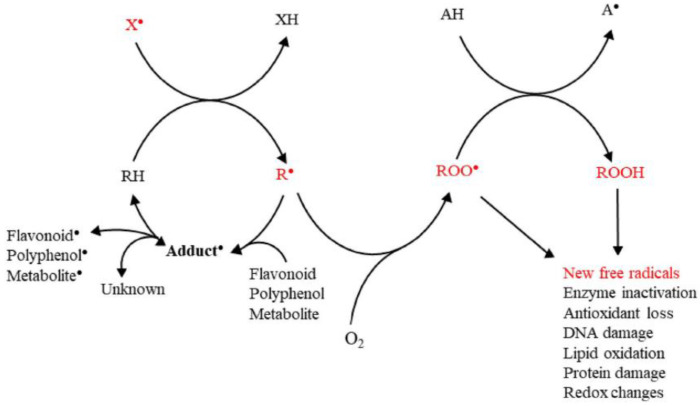
Schematic depiction of the action of highly reactive free radical species and their inhibition by phenolic compounds. Reprinted (adapted) with permission from [66].

**Figure 4:**
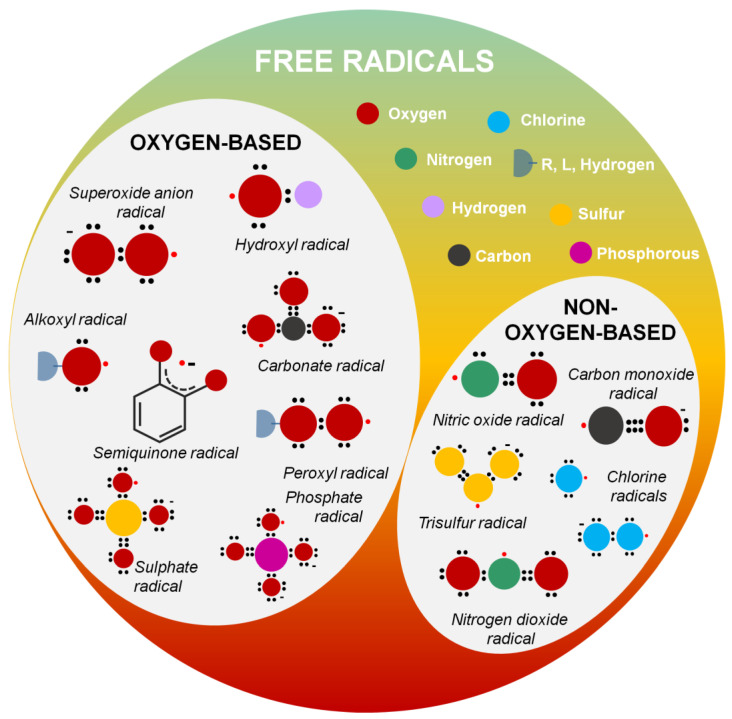
Free radicals are highly reactive species. It is a general term that describes species that bear at least one unpaired electron and includes oxygen-centered (some of the ROS) and non-oxygen-centered radicals such as nitrogen-centered (some of the RNS), carbon-centered, sulfur-centered, phosphorous-centered, and halogen-centered structures. Antioxidants counterbalance the severe cell damage when free radicals react with biological molecules.

Free radicals are highly reactive species that can be neutralized by phenolic compounds and other types of antioxidants (see Figure 3 and Figure 4) [64]. It is a general term that describes species that bear at least one unpaired electron, and includes oxygen-centered (some of the ROS) and non-oxygen-centered radicals, such as nitrogen-centered (some of the RNS), carbon-centered, sulfur-centered, phosphorous-centered, and halogen-centered structures. Several pathways can generate free radicals, including homolytic and heterolytic cleavage of a molecule or redox-mediated reactions [3]. They can be positively, negatively, or neutrally charged. To become stabilized, their free electron has to be paired with another belonging to an atom or molecule in the surrounding environment [3]. In living organisms, free radicals are produced as natural intermediates of biochemical reactions, and play a crucial role in the physiological cell function. They can, moreover, be formed by extrinsic factors (environmental pollution, smoking, unhealthy diet, alcohol consumption, stress, or exposure to ultraviolet (UV) light) [65]. Finally, exposure to low-wavelength electromagnetic radiation can lead to hydroxyl radicals generation due to water splitting in the body [2].

i. *Oxygen-centered radicals*

According to Table 1, among the most common oxygen-centered free radicals, namely radical species where the unpaired electron is located in the oxygen atom, are the hydroxyl radicals (^•^OH) [64], the superoxide anion radicals (^•^O_2_^−^) [64], the hydroperoxyl (HO_2_^•^) radical [64], the alkoxyl (RO^•^)[64,67], and peroxyl radical (ROO^•^) [64], lipid alkoxyl (LO^•^)[64,67] and lipid peroxyl (LOO^•^) radicals [64], semiquinone (SQ^•−^) [68] radicals, and carbonate (CO_3_^•−^) radicals [69,70]. Similarly to alkoxyl radicals, phenoxyl radicals (e.g., tyrosyl radical, Tyr^•^) are common, especially in biological systems [71,72]. Moreover, sulfate radicals (SO_4_^●−^) [73] are generated via the activation of persulfates, such as peroxydisulfate (PDS, S_2_O_8_^2−^) and permonosulfate (PMS, HSO_5_^−^), characterized by the presence of an O–O bond (similar to hydrogen peroxide) [74]. Sulfate radicals SO_4_^●−^ have a higher redox potential than hydroxyl radicals (OH^●^) [73,74]. Finally, phosphate radicals (PO_4_^2−●^) are intermediate radical species with an oxidative ability (Figure 1, Table 1) [67].

ii. *Nitrogen-centered radicals*

Ordinary nitrogen-centered radicals include nitric oxide radical (^•^NO) [75] and nitrogen dioxide radical (^•^NO_2_) [64]. When nitric oxide (^•^NO) radicals and superoxide anion (^•^O_2_^−^) radicals react, peroxynitrite (ONOO^−^) is generated (Figure 1, Table 1) [73].

iii. *Carbon-centered radicals*

Carbon-centered radicals are among the most common radical species. The oxygen-centered radical and aliphatic carbon reactions can form a carbon-centered radical [76]. They can generally be categorized as aromatic carbon-centered radicals, aliphatic carbon-centered radicals, and carbon-centered radicals with an adjacent oxygen atom [76]. When the carbon monoxide molecule acquires an electron, the radical anion (CO^●−^) is formed to further react with CO^●−^ or CO, forming carbon–carbon bonds (Figure 1, Table 1) [77].

iv. *Sulfur-centered, phosphorous-centered, and halogen-centered radicals*

Sulfur-centered, phosphorous-centered, and halogen-centered radicals are typical moieties that must be counterbalanced. Characteristic examples include the trisulfur radical (S_3_^•−^) [78] or chlorine radicals [79] (Cl^•^, Cl_2_^•−^) (Figure 1, Table 1).

Reactive oxygen species (ROS) is a term used to describe radical and non-radical reactive forms of oxygen. Increased concentration of ROS, either due to cell malfunction or extrinsically induced, can lead to severe damage due to oxidative stress [80]. Among the most common radical ROS species is the superoxide anion radical (^•^O_2_^−^, half-life of 1–1000 µs or 10–6 s) [64] that reacts with H^+^ to produce the more reactive and harmful to cell membranes hydroperoxyl (HO_2_^•^) radical. Of high interest are also hydroxyl radicals (^•^OH) [81], with a half-life of approximately 10^−10^ s [64], that possess the highest one-electron redox potential of all the relevant ROS, which increases their capacity to endanger the biological processes of the cells [82,83]. Alkyl peroxyl radicals (ROO^•^) are responsible for polyunsaturated fatty acids’ peroxidation, and organic materials’ autoxidation reactions [58]. On the other hand, hydrogen peroxide (H_2_O_2_) is an important, stable, non-radical ROS [64]. Other species of non-radical ROS include singlet oxygen (^1^O_2_, half-life 10^−6^ s) [11], [64], that can be formed upon oxidation of vitamin E [84], ozone (half-life a few seconds) [64], stable organic peroxide (RCOOH) [64], and, finally, hypochlorous acid (HOCl) [64] and hypobromous acid (HOBr) [64], which are stable for a few minutes.

Reactive nitrogen species (RNS) is a term used to describe radical and non-radical reactive forms of nitrogen. RNS include several nitric oxide-derived compounds, such as nitroxyl anion, nitrosonium cation (NO^+^), higher oxides of nitrogen, S-nitrosothiols (RSNOs), or dinitrosyl iron complexes excluding NO_3_^−^. Ordinary nitrogen-centered radicals include nitric oxide radical (^•^NO) and nitrogen dioxide radical (^•^NO_2_) [64]. Non-radical forms of RNS include dinitrogen trioxide (N_2_O_3_), dinitrogen tetroxide (N_2_O_4_), nitrite (NO_2_^−^), nitronium cation (NO_2_^+^), nitrosoperoxycarbonate anion (ONOOCO_2_^−^), nitrous acid (HNO_2_), peroxynitrous acid (ONOOH), nitroxyl (HNO), nitryl chloride (Cl-NO_2_), alkyl peroxynitrites (ROONO), and peroxynitrites (ONOO^−^). Like ROS, RNS are crucial components in maintaining physiological cell functions. An increase in the concentration of RNS can lead to cell injury and death by inducing nitrosative stress. During pathologic conditions, ^•^NO reacts with other species, such as the superoxide anion (O_2_^−^), through an enzyme-independent mechanism, and can become very harmful to the cell due to the formation of peroxynitrite (ONOO^•^). This strong oxidant reacts with most biological molecules, causing severe cell damage [75].

### 3.2. Mechanisms of Antioxidant Activity

The effective action of antioxidants can be influenced by many factors, such as their structural characteristics, the concentration and temperature at which they act, the type of substrate they must deal with, the system’s physical state, and many others. The intrinsic activity of an antioxidant towards free radicals and other reactive oxygen species and, therefore, its antioxidant activity, is determined by its chemical structure plus its micro-environment. Finally, a determining factor for the effectiveness of an antioxidant is its concentration [85].

The first step in developing and evaluating an antioxidant system’s efficiency is identifying its action mechanism. Although the common purpose is neutralizing a damaging moiety, e.g., ROS, in most cases, this can be achieved through many pathways. In their recent review, Shah et al. [1] propose the pertinent classification that nanoantioxidants can act either as damage-preventive agents or chain-breaking antioxidants. Damage-preventive mechanisms include enzyme-mimicking activity, such as catalase (CAT)-mimetic, superoxide dismutase (SOD)-mimetic, or glutathione peroxidase (GPx)-mimetic [1]. Chain-breaking antioxidants interfere in specific radical-initialized chain reactions, such as autoxidation reactions [62].

**Figure 5:**
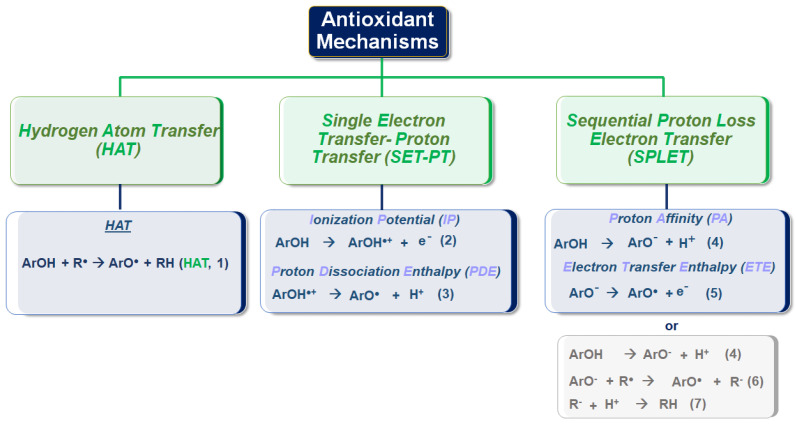
Antioxidant activity can be generally categorized in the three mechanisms depicted above: (**i**) hydrogen atom transfer (HAT), proton-coupled electron transfer (PCET), (**ii**) single electron transfer-proton transfer(SET-PT), and (**iii**) sequential proton loss electron transfer (SPLET).

Antioxidants are capable of scavenging free radicals using three mechanisms [86]: (i) hydrogen atom transfer (HAT), proton-coupled electron transfer (PCET), (ii) single electron transfer-proton transfer (SET-PT), and (iii) sequential proton loss electron transfer (SPLET) (see Figure 5). The HAT and PCET mechanisms are the primary mechanisms, while SPLET can be viewed as a subset of the main mechanisms. Theoretical tools, i.e., such as Density Functional Theory (DFT) calculations, can provide key-insights [16]. The work of Mayer et al. [16] categorizes the mechanisms that involve a single-step donation of an electron and a proton into hydrogen atom transfer (HAT) and proton-coupled electron transfer (PCET). Their work exemplifies a {benzyl/toluene} reaction scheme and a {methoxyl/methanol} scheme that can proceed via a HAT mechanism, or a {phenoxyl/phenol} that can proceed via the PCET mechanism [16].

### 3.3. Hydrogen Atom Transfer (HAT) and Proton-Coupled Electron Transfer (PCET) Pathways

**Figure 6:**
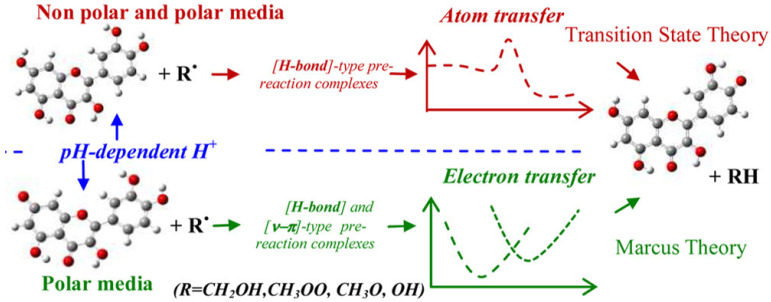
Schematic depiction of the hydrogen atom transfer (HAT) mechanism. Reprinted (adapted) with permission from [89]. Copyright 2023 American Chemical Society.

**Figure 7:**
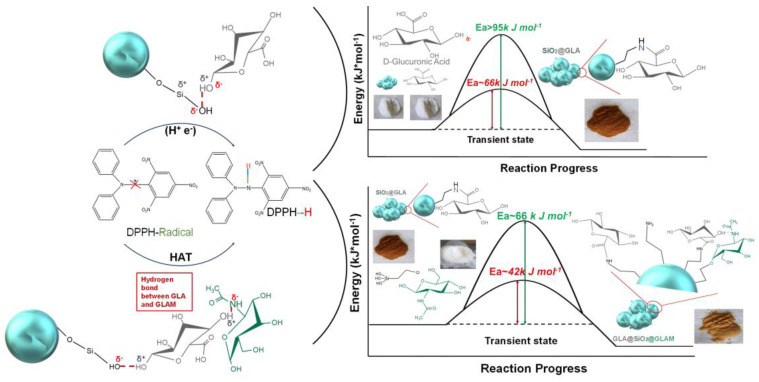
Schematical depiction of the hydrogen-atom-transfer (HAT) mechanism between a phenolic OH of grafted GLA-GLAM to a DPPH radical. Reprinted (adapted) with permission from [15]. Copyright 2023 American Chemical Society.

Hydrogen atom transfer (HAT) [87] is one of the most common and crucial antioxidant mechanisms in chemical and biological processes, often operating in phenolic and polyphenolic antioxidant systems. During HAT (see Figure 6), the antioxidant donates {one proton plus an electron} that are transferred together as a single hydrogen atom to neutralize a free radical [16]. The HAT mechanism has additionally been described in detail in our group’s recent work, where nanohybrid structures have been developed and evaluated as antioxidants (see Figure 7) [15,29,88]. Similar to the HAT mechanism, the Proton-coupled electron transfer (PCET) mechanism describes a proton and electron transfer between different orbitals [16]. It is the antioxidant mechanism used by vitamin E (tocopherol) [16].

HAT reactions [89] (see Reaction 1) are electronically adiabatic because the electron transfer distance is relatively short and involves an electronic surface. At a fundamental level, HAT reactions are characterized by proton and electron transfer [90], and they are the most straightforward class of PCET processes. The free radical is stabilized to neutral species, while the antioxidant is converted to a free radical [91]. Phenolic molecules, for instance, provide an H-atom to a free radical substrate to produce a non-radical substrate (RH, ROH, or ROOH) while gaining a free radical [3]. They involve the transfer of an electron and a proton from one reactant to another, and are always in the same kinetic step (see also Figure 6).

ArOH + R^●^ → ArO^●^ + RH ((HAT, **Reaction 1**)

Notably, chemical energy and fuel production typically involve HAT or PCET reactions. An example is the HAT key step in the combustion of hydrocarbons (H/C) [87]. PCET processes participate in many other chemical reactions of antioxidants, such as vitamins C and E, in the environment’s redox dissolution of metal oxides. A critical step of these reactions is the {group-transfer} of {electrons plus protons}. Coordinated {e^−^/H^+^} transfer reactions have received experimental and theoretical attention because they are the simplest PCET process [92]. The differences in activation energies are usually expressed as the differences in the so-called bond dissociation enthalpies (BDE) [92]. The lower the BDE value, the easier the dissociation and detachment of the H-atom [93].

### SET-PT Single Electron Transfer-Proton Transfer

The second possible mechanism of action of antioxidants is a single electron transfer-proton transfer (SET-PT) process. In this process, the electron transfer step is followed by the H^+^ transfer (SET-PT) mechanism in two steps. In the first step (ArOH **→** ArO^●+^), a cationic [ArOH^●+^] radical is formed. In the second step, deprotonation of the cationic radical occurs (ArOH^●+^ **→** ArO^●^ + H^+^).

The ionization potential (Reaction 6) and the proton dissociation enthalpy (Reaction 7) represent the enthalpies of the SET-PT mechanism [93]. The ionization potential (IP) is the enthalpy required to detach the electron. The change in enthalpy of the reaction is called the proton dissociation enthalpy (PDE) [94].

ArOH → ArO^●+^ + e^−^ Ionization Potential (IP) (**Reaction 2**)

ArOH^●+^ → ArO^●^ + H^+^ Proton Dissociation Enthalpy (PDE) (**Reaction 3**)

### Sequential Proton-Loss Electron Transfer (SPLET)

Another mechanism is sequential proton loss and electron transfer (SPLET). The enthalpy of the first step reaction corresponds to the proton affinity (PA) (Reaction 4) of the phenoxide anion. In the second step, electron transfer takes place from the phenoxide anion to the radical, and thus a phenoxy radical is formed. The enthalpy of the reaction in this step is called the electron transfer enthalpy (ETE) (Reaction 5). From the side of antioxidant activity, the net result of the SPLET mechanism is the same as the HAT mechanism and the SET-PT mechanism, i.e., the transfer of the hydrogen atom to the free radical. The enthalpies of the reactions (BDE, IP, PA) related to the three mechanisms mentioned above are significant in evaluating the antioxidant activity [93].

Sequential proton loss electron transfer (SPLET) can occur in two or three steps. The two-step mechanism involves the loss of a proton (Reaction 4); thus, the polyphenolic anion undergoes electron transfer (Reaction 5). The SPLET mechanism is preferred when the anion (ArO^−^) is stable enough to allow electron transfer before re-protonation.

ArOH → ArO^−^ + H^+^ Proton Affinity (PA) **(Reaction 4)**

ArO^−^ → ArO + e^−^ Electron Transfer Enthalpy (ETE) **(Reaction 5)**

This three-step process can be described in reactions 4 (the same as the two-step mentioned above mechanism), 6, and 7 [89].

ArOH → ArO^−^ + H^+^ (**Reaction 4**)

ArO^−^ + R^●^ → ArO^●^ + R^−^
**(Reaction 6)**

R^−^ + H^+^ → RH (**Reaction 7**)

These three mechanisms described above, namely hydrogen atom transfer (HAT)/proton-coupled electron transfer (PCET), single electron transfer-proton transfer (SET-PT), and sequential proton loss electron transfer (SPLET), present similar thermodynamic equilibrium since the reactants and products are the same (ΔG_PCET_ = ΔG_ET-PT_ = ΔG_SPLET_). The competition between the different mechanisms is governed by the kinetics of the rate-limiting step of each mechanism (atom transfer for HAT/PCET and electron transfer for both ET-PT and SPLET). Under increasing solvent polarity, the ET-PT mechanism is disfavored, i.e., due to the instability of the cationic phenolic radical (ArOH^●+^). In a non-polar environment, e.g., such as lipid bilayer membranes, the PCET process is the prevailing active process. In other words, the PCET process is the primary process that breaks the lipid peroxidation chain reaction [89]. The only way for reactions that take place at low pH values, such as stomach reactions, is to adopt the PCET process [89].

## 4. Evaluation of the Antioxidant Activity

Effective detection and quantification of the different radical species play a crucial role in radical chemistry and the accurate evaluation of antioxidant capacity. There is a need for precise-quantitative methods to measure antioxidant activity effectively. Herein, we review the antioxidant evaluation methods referred to in the bibliography, depending on the type and specific antioxidant application, to provide a summarized guide.

**Figure 8:**
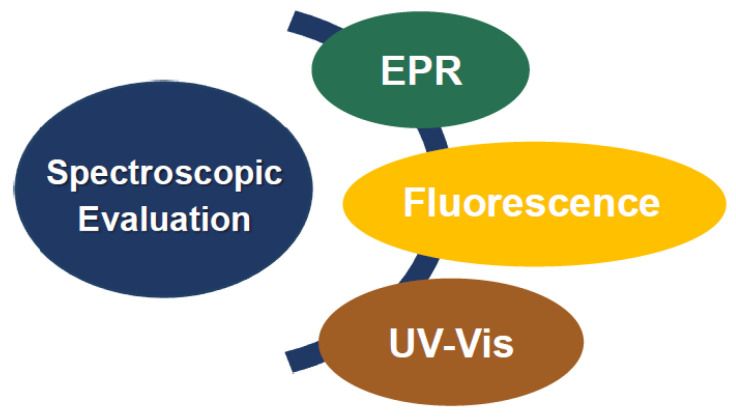
Spectroscopy plays a crucial role in the determination of antioxidant activity. Spectroscopic evaluation can be achieved through (**i**) electron paramagnetic resonance (EPR), (**ii**) ultraviolet-visible (UV–Vis), and (**iii**) fluorescence spectroscopy. EPR spectroscopy enables the direct detection of species that bear at least one single electron. In contrast, different assays can be used to evaluate antioxidant activity through fluorescence and UV–Vis spectroscopy.

Spectroscopy offers valuable tools for the determination of antioxidant activity. Among others, spectroscopic evaluation can be achieved through (i) electron paramagnetic resonance (EPR), (ii) ultraviolet–visible (UV–Vis), and (iii) fluorescence spectroscopy. The specific experimental methodologies for evaluating the antioxidant activity can be divided into direct methods for detecting a species, such as radical detection by EPR, and indirect methods (e.g., DPPH method), where a physicochemical alteration due to the radical scavenging mechanism is being detected. The chosen evaluation method is mainly based on the specific antioxidant mechanism (Figure 8). For example, the HAT mechanism can effectively be studied utilizing the DPPH radical method [15].

### 4.1. Evaluation Based on Electron Paramagnetic (EPR) Resonance Spectroscopy

**Figure 9:**
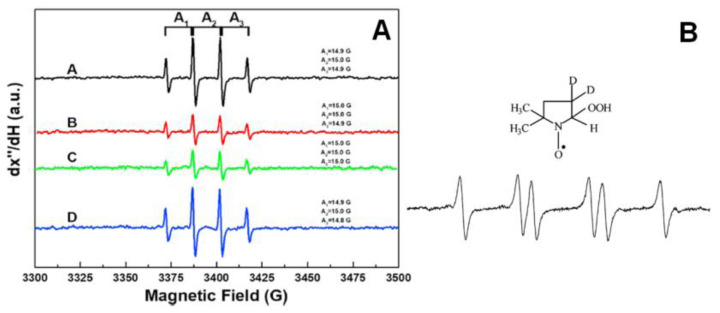
To monitor radical species with very short half-life times, such as hydroxyl (^•^OH) or superoxide (^•^O_2_^−^) radicals, spin-trap molecules such as 5,5-dimethyl-pyrroline N-oxide (DMPO) are needed. Figure 9 depicts the typical signals of (**A**) DMPO-^•^OH adduct, where the characteristic hyperfine splitting of the hydroxyl quartet (A_1_, A_2_, A_3_) is displayed. (A-black line) no antioxidant; (B-red line) in the presence of Rosmarinic Acid; (C-green line) in the presence of RC; (D-blue line) in the presence of RCG. (**B**) DMPO-^•^OOH adduct. Reprinted (adapted) with permission from [95,96]. Copyright 2023 American Chemical Society/Elsevier.

Electron Paramagnetic Resonance (EPR) Spectroscopy is a highly sensitive spectroscopic technique for directly detecting and quantifying species with one or more single electrons. For completeness, we clarify that electron spin resonance (ESR) refers to the cases where there is no major spin-orbit coupling contribution, i.e., typical for s- or p-radicals, while EPR is more general since it includes the case of significant spin-orbit couplings, i.e., typical for *d*-electrons in metals or S-based radicals [69], [73]. EPR spectroscopy detects free radicals that lead to oxidative stress and cell damage. To monitor radical species with very short half-life times, such as hydroxyl (^•^OH) or superoxide (^•^O_2_^−^) radicals, spin-trap molecules such as 5,5-dimethyl-pyrroline N-oxide (DMPO) are needed. The typical detectable signals of DMPO-^•^OH and DMPO-^•^OOH are presented in Figure 9 [95,96]. EPR provides quantitative information on the radical species based on comparison with an appropriate spin standard such as DPPH [61]. The antioxidant capacity can be chemically quantified, and can be considered directly proportional to the decrease in the characteristics of the experimental EPR signal of the radical in comparison to a control spectrum (e.g., area, intensity, etc.).

### 4.2. Evaluating Antioxidant Activity via Fluorescence Spectroscopy

#### 4.2.1. Radical Trapping Antioxidant Parameter (TRAP) Assay

The total antioxidant radical trapping parameter (TRAP) measures the ability of antioxidants to control and regulate the anti-peroxidation reaction using (2,2-azobis-2-methyl-propanimidamide)dihydrochloride (AAPH), and 2,2-azobis (2-amidinopropane)dihydrochrolide (ABAP), as sources of radicals [97]. This method is based on the protection provided by antioxidants, during a controlled reaction, through the fluorescence decay of R-phycoerythrin (R-PE) [98]. R-phycoerythrin (R-PE), the brightest fluorescent pigment ever identified and isolated from the red alga Graciloria, is used as a fluorescence detector [99]. The progress of the reaction of R-PE and AAPH is monitored fluorometrically (λ_ex_ = 495 nm, λ_em_ = 575 nm). The HAT mechanism determines the TRAP, while a fluorometer monitors oxidation. The antioxidant capacity of a compound can be determined by comparing the extension of the delay time for the oxidant probe to appear, always in the presence of a sample, to the corresponding times for Trolox [97]. During the reactions of this method, the fluorescence of R-phycoerythrin is quenched by ABAP (2,2′-azo-bis(2-ami-dinopropane) hydrochloride), which is a radical activator [98].

#### 4.2.2. Oxygen Radical Absorbance Capacity (ORAC) Assay

**Figure 10:**
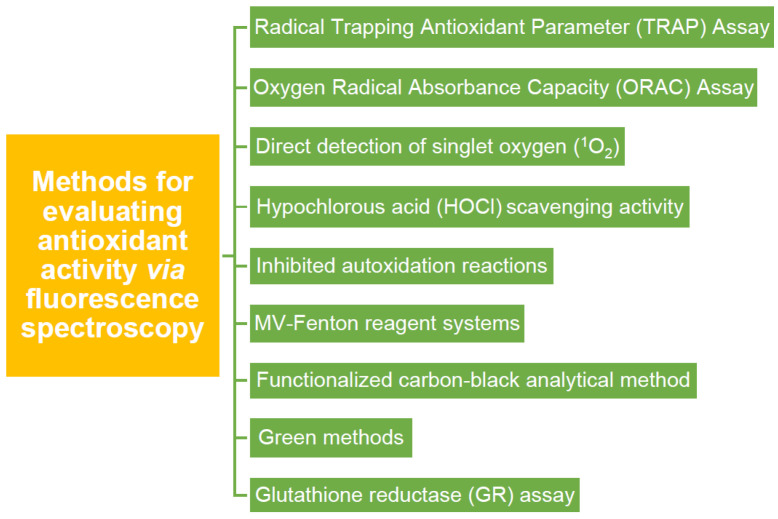
Multiple methodologies are referred to in the literature for evaluating antioxidant activity using fluorescence spectroscopy.

The ORAC method, i.e., the oxygen radical absorption capacity method, is a revolutionary method that is becoming increasingly useful worldwide for measuring antioxidant capacity in biological samples and food. The specific method is based on inhibiting the induced peroxy-radical oxidation through the thermal decomposition of azo compounds, such as 2,2′-azobis(2-aminodino-propane) dihydrochloride (AAPH). The role of antioxidants in this reaction is to suppress, through the HAT mechanism, the oxidative degradation of the fluorescein signal (fluorescence). The ORAC method is characterized by its uniqueness in combining both the inhibition time and the degree of inhibition in a single quantity. In this method, either β-phycoerythrin (β-PE) or fluorescein is used as a target molecule, as a fluorescence detector, and the fluorescence that decreases in the presence of free radical scavengers, i.e., antioxidants, is measured. This method measures the loss of fluorescence over time due to the peroxy radical formed by an initiator’s breakdown and cleavage, such as bis-azide, AAPH, (2,2,-azobis- 2-methyl-propanimidamine) dihydrochloride at 37 °C. The decrease in fluorescence is monitored visually [99]. One of the most well-known antioxidants used in this method is Trolox, which is soluble in water and a positive regulator inhibiting fluorescence. The fluorescence signal is measured over thirty minutes, with excitation at 485 nm, emission at 538 nm, and cutoff at 530 nm. During the application of the method, the antioxidant concentration in the sample is proportional to the fluorescence intensity. It is evaluated by comparing the net area under the curve with the known values given by Trolox as a standard [99].Other evaluation methods referred to in the bibliography include the direct detection of singlet oxygen (^1^O_2_) [28], hypochlorous acid (HOCl) scavenging activity [100], different types of inhibited autoxidation reactions [58], or MV-Fenton reagent systems [101]. An analytical method has been developed based on the functionalization of an antioxidant in Carbon Black [102]. Additionally, greener evaluation methods have been proposed [103]. Finally, the glutathione reductase (GR) assay is a commonly used antioxidant evaluation method [104]. Everything mentioned in the text is summarized in Figure 10, where the methods for evaluating the antioxidant activity of materials through fluorescence spectroscopy are mentioned.

### 4.3. Evaluating Antioxidant Activity via UV–Vis Spectroscopy

Among the methods that incorporate UV–Vis spectroscopy as a primary evaluation tool are ^●^OH radicals, DPPH^●^, ABTS^●+^, ^●^NO, as well as H_2_O_2_ scavenging assay.

#### 4.3.1. Evaluating ^●^OH Radicals Scavenging Capacity via Assay

An antioxidant’s hydroxyl radical (^●^OH) scavenging capacity can be evaluated using a specific assay that includes solutions of DMPO (200 mM) and H_2_O_2_ (10% *v*/*v*) prepared in MilliQ water. For instance, to evaluate the ^●^OH RSC of Morin, the antioxidant was dissolved in a glycerin solution 10% *v*/*v*, including a small quantity of ethanol as co-solvent (0.2% of the final volume) to a final concentration of 0.11 mM. A control solution, with DMPO 200 mM, hydrogen peroxide 10%, and glycerin 10%, was additionally prepared. DMPO-HO^•^ adducts can be thereby detected [32].

#### 4.3.2. Evaluating DPPH^●^ Radicals Scavenging Capacity

**Figure 11:**
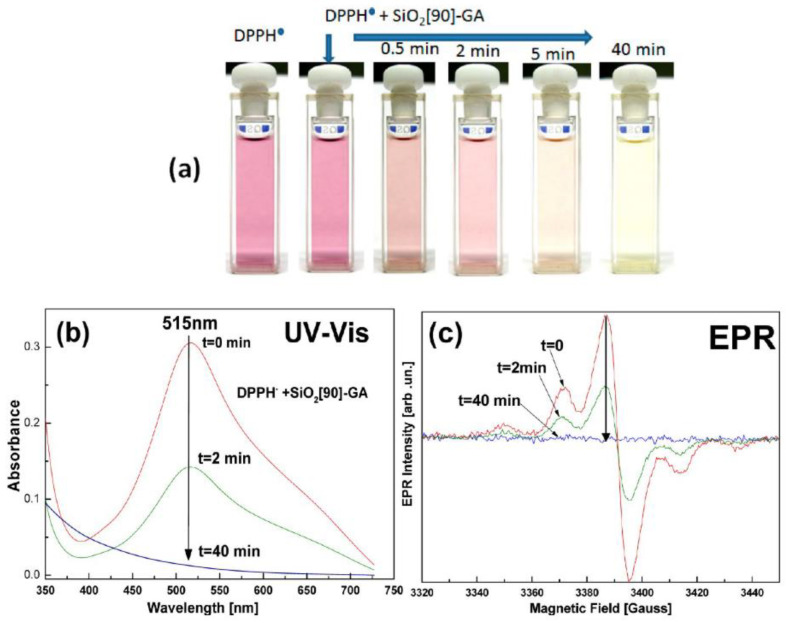
UV–Vis (**b**) and EPR (**c**) spectroscopic analysis of the DPPH radical scavenging by nanoantioxidants. The absorption from the visible region disappears (**b**), and the color of the solution changes from purple to pale yellow (**a**). Reprinted (adapted) with permission from [88]. Copyright 2023 American Chemical Society.

2,2-diphenyl-1-picrylhydrazyl (DPPH) is a stable molecule that bears a nitrogen-centered free radical. It is particularly interesting in research regarding antioxidants, since its color decays from violet to yellow upon reduction and does not need to be generated before analysis [105]. The stability of the radical makes the DPPH method easy, accurate, and cost-effective for evaluating the radical scavenging activity [106]. Through this specific radical, the ability of certain compounds to act as free radical scavengers or hydrogen donors can be tested, and their antioxidant activity evaluated [107]. In the UV–Vis spectrophotometer, a peak is generated by the π−π* transitions with a significant contribution from the non-bonded electron pair in the visible region, λ_max_ around 515 nm. At the same time, the extinction coefficient is slightly solvent-dependent. The DPPH radical has the property of changing the orientation of the free electron through the molecule so that it does not dimerize, as it would with most other free radicals [98]. This radical can be reduced by adding a hydrogen atom from a hydrogen donor compound, such as the antioxidant, thus forming a DPPH-H hydrazine. With the formation of hydrazine, the absorption from the visible region disappears, and the color of the solution changes from purple to pale yellow (see Figure 11) [108]. The rapid decrease in absorption of the DPPH radical determines the capacity of the antioxidant. A stronger antioxidant implies a more rapid decrease in absorption. Thus, the antioxidant can better act through the HAT mechanism [109].

#### 4.3.3. ABTS^●+^ Radicals Scavenging Capacity (ABTS^●+^ Method) or Trolox Equivalent Antioxidant Capacity (TEAC/ABTS^●+^)

The scavenging of 2,2-azinobis-(3-ethylbenzothiazoline-6-sulphonate) radical cation (ABTS^●+^), also called ABTS^●+^ cation decolorization assay, allows measurement of the antioxidant capacity of compounds and helps distinguish between additive and synergistic effects [98]. This method is based on the interaction between an antioxidant, which in this case is Trolox, with the radical cation ABTS^●+^, which has a characteristic blue-green color with maximum absorption occurring at 645, 734, and 815 nm [97]. ABTS^●+^ is a stable radical, soluble in both water and organic solvents, and helps to determine the antioxidant capacity of both hydrophilic and lipophilic compounds. The ABTS^●+^ method has good repeatability and is simple to perform. The results are related to a standard antioxidant compound exhibiting a different kinetic behavior during the reaction [97]. The ABTS^●+^ cationic radical is not commercially available and must be generated before use. It can be produced by the reaction of an oxidizing agent, such as sodium or potassium persulfate or MnO_2_ with 2,2′-azino-bis(3-ethylbenzthiazoline-6-sulphonic acid) ABTS, to form the ABTS^●+^ radical showing blue-green color [99]. After adding an antioxidant that acts as a hydrogen atom donor, the blue-green cationic radical ABTS^●+^ is reduced. Its reduction is measured by the decrease in the characteristic absorption spectrum’s prominent peaks. Lipophilic and hydrophilic compounds and food extracts can be used as antioxidants, including flavonoids, hydroxycinnamate, and carotenoids [97,99]. The best-known method today is the TEAC-II method, which is considered better than the TEAC-I one, which uses metmyoglobin-H_2_O_2_ to generate ^●^OH, where it will then react with ABTS to generate the radical cation. With the TEAC-II method, firstly, there is a direct formation of the cationic radical ABTS^●+^ without the involvement and participation of an intermediate radical; secondly, the cationic radical is formed before the addition of the antioxidant to the system [99].

#### 4.3.4. Hydrogen Peroxide Scavenging (H_2_O_2_) Assay

H_2_O_2_ is generated in vivo by various oxidative enzymes, under physiological conditions, catalyzed by superoxide dismutase or by the decomposition of the peroxide radical [97]. There are several ways hydrogen peroxide can enter the body, such as by inhaling vapors, as well as through the eyes or skin. H_2_O_2_ can rapidly decompose into oxygen and water, which causes the production of hydroxyl radicals (^●^OH), responsible for lipid peroxidation and DNA damage [98]. One of the most common methods for evaluating the scavenging capacity against this molecule is based on the intrinsic absorption of H_2_O_2_ in the UV region. The ability to scavenge the H_2_O_2_ of an extract is directly related to its antioxidant activity. This method involves the in vitro generation of the hydroxyl radical using the [Fe^3+^/ascorbic acid/EDTA/H_2_O_2_] system via the Fenton reaction [97].

#### 4.3.5. Nitric Oxide Radical (^●^NO) Scavenging Assay

The nitric oxide (^●^NO) radical contains a non-bonded electron and exhibits significant activity on specific proteins and other free radicals [97]. The *^●^*NO is regenerated in biological tissues by a specific nitric oxide capable of metabolizing arginine to citrulline, with a five-electron oxidative reaction, to form a *^●^*NO radical [98]. In this method, the in vitro inhibition of the *^●^*NO provides a measure of antioxidant activity. In buffered saline, the radical of ^●^NO is produced from nitroprusside (sodium nitroprusside). The scavenging ability of this radical can also be applied to in vitro purifications, where nitric oxide remains after the reaction with the antioxidant and is measured as nitrite. Because *^●^*NO may be formed during the reaction, it must be reduced to nitrite before determining antioxidant capacity. In the presence of antioxidants capable of scavenging the radical, the absorbance of the chromophore is evaluated at 546 nm. Antioxidant capacity is expressed as the percent reduction of nitric oxide [97].

#### 4.3.6. Ferric Reducing Antioxidant Power (FRAP) Assay

Benzie and Strain presented the total antioxidant activity measured during the ferric-reducing antioxidant power (FRAP) assay [92]. Essentially, this method measures the ability of an antioxidant to reduce iron (ferric acid). More specifically, the iron complex 2,3,5-triphenyl-1,3,4-triazo-2-azoniacyclopenta-1,4-diene chloride (TPTZ) is reduced to the Fe (II) form at low pH [98]. This colorimetric method uses antioxidants as reducing agents in redox reactions [99]. It is a simple and inexpensive spectrophotometric technique. The reaction occurs via electron transfer, and FRAP values are calculated by measuring the increase in absorbance at 593 nm. They are related to a standard solution of iron Fe (II) ions or a standard antioxidant solution (e.g., ascorbic acid). At low pH (3.6), the reduction of iron (III) tripyridyl triazine (Fe III TPTZ) to the complex with the ferrous form (Fe II), which has an intense blue color, can be monitored by measuring the change in absorbance at 593 nm. The total reducing power is related to the change in absorbance with electron transfer through the presence of antioxidants in the reaction mixture. Ferrous sulfate solution is used as a standard solution [99].

#### 4.3.7. Inhibiting Autoxidation Reactions/Lipid Peroxidation Inhibition Assay

Lipid peroxidation is a well-known mechanism of cell injury in plants and animals, and is used as an indicator of oxidative stress in cells and tissues [99]. It is an auto-catalytic process that leads to cell death [98]. Lipid peroxidation is unstable and decomposes into a complex series of compounds, including reactive carbonyl compounds. This method is based on the reaction of a chromogenic reagent, N-methyl-2-phenylindole, with MDA and 4-hydroxyalkenals at 45 °C. One molecule of either MDA or 4-hydroxyalkenals reacts with two molecules of N-methyl-2-phenylindole to produce a stable chromophore (carbocyanine dye) with maximum absorption at 568 nm [99].

**Figure 12:**
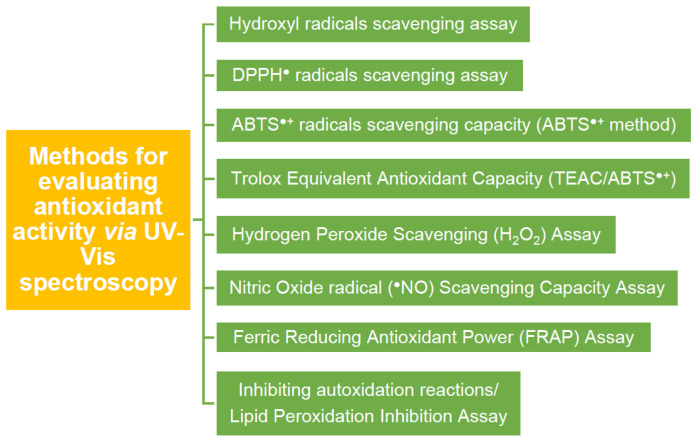
Multiple methodologies are referred to in the literature for evaluating antioxidant activity using UV–Vis spectroscopy.

All the methods referred above, used to evaluate an antioxidant mainly through UV–Vis spectroscopy, can be seen in Figure 12.

### 4.4. Expressing Antioxidant Capacity

In addition to the different evaluation methods, there are different ways to express antioxidant activity. Among them, antioxidant activity is expressed directly in terms of concentration (mg of antioxidants or radicals/L) [110] or amounts expressed in chemical terms (moles of antioxidants/moles of scavenged radicals) [15]. Results can also be focused on directly detecting generated free radicals from a material that can be expressed as moles of radicals/mg of material [61].

It is common to state antioxidant activities with terms of half-maximal effective concentration (EC_50_) or inhibitory concentration (IC_50_)_,_ namely, the concentration required to obtain a 50% radical scavenging. EC_50_ values are often utilized to express antioxidant capacity between different compounds [110]. The strength of an antioxidant can be expressed by the lower value of EC_50_ (or IC_50_) [111]. In the case of the DPPH evaluation, the IC_50_ measures the reduction of the initial concentration of DPPH when it reaches half of it, i.e., 50%, after the addition of the antioxidant [109]. A compound’s antioxidant capacity can generally be expressed as radical scavenging capacity (% RSC). The % RSC can be estimated by Equation (1) [45].

(1) $$\%\;\mathrm{RSC}=\left.\left(\;\frac{A0-A1}{A0}\right.\right)\times 100$$

where A_0_ is the initial absorption, while A_1_ is the absorption after the reaction.

Other researchers define antioxidant activity utilizing physicochemical parameters such as the *n* value, which can be calculated in three ways [88]:

(2) $$n_{\mathrm{total}}:\;n_{total}=\frac{DPPH_{total\;scavenged}}{{\left[Antioxidant\right]}_{0}}\;$$

(3) $$n_{\mathrm{fast}}:\;n_{fast}=\frac{moles\;of\;DPPH\;radicals\;scavenged\;via\;HAT\;reactions}{{\left[Antioxidant\right]}_{0}}\;$$

(4) $$n_{\mathrm{slow}}:\;n_{slow}=\frac{moles\;of\;DPPH\;radicals\;scavenged\;via\;Secondary\;reactions}{{\left[Antioxidant\right]}_{0}}\;$$

Moreover, the physicochemical study can be expanded using the Arrhenius study, since the first approach to determining the rate of chemical reactions was made through the reaction rate law by Svante Arrhenius [112]. The dependence of the reaction rate on temperature is expressed through the Arrhenius equation (Equation (5)) [112].

(5) $$k=A\times e^{-E_{a}/RT}\;$$

where, *k*: is the rate constant of the reaction, and is calculated from the slope of the curve, *T*: is the temperature in Kelvin, *A*: is a pre-exponential factor, a constant for any chemical reaction that determines the rate of the collision frequency in the correct direction and, in addition, is a factor that has a weak dependence on the rate of the reaction rate, *Ea*: is the activation energy of the reaction and is measured in (Joules/mole) and *R* symbolizes the global gas constant in (J/Kmol) [112]. For the easier calculation of the activation energy, the Arrhenius equation can be written in another way, such as in logarithmic form (Equation (6)):

(6) $$\mathrm{lnk}=(-E_{a}/R)\times (1/T)+\;\mathrm{lnA}\;$$

The differences in activation energies are usually expressed as the differences in bond dissociation enthalpies (BDE) [92]. The lower the value of the decomposition enthalpy (Equation (7)), the easier the dissociation and the detachment of the phenolic -OH [93]. The equation linking activation to switching is shown below (Equation (7)):

(7) $$E_{a}=\alpha \mathrm{BDE}\left(\mathrm{ArOH}\right)+\;\beta \;$$

where α and β are constants, the constant α depends on the transition state’s position during the reaction, while ArOH symbolizes the phenolic molecules.

Finally, the antioxidant activity of a studied system is often expressed in equivalents of standard reference antioxidant systems; thus, terms such as GA equivalent (GAE) [36] or ascorbic acid equivalent antioxidant activity are commonly encountered.

## 5. Optimizing Antioxidant Systems by Biomimetic Nanoengineering

**Figure 13:**
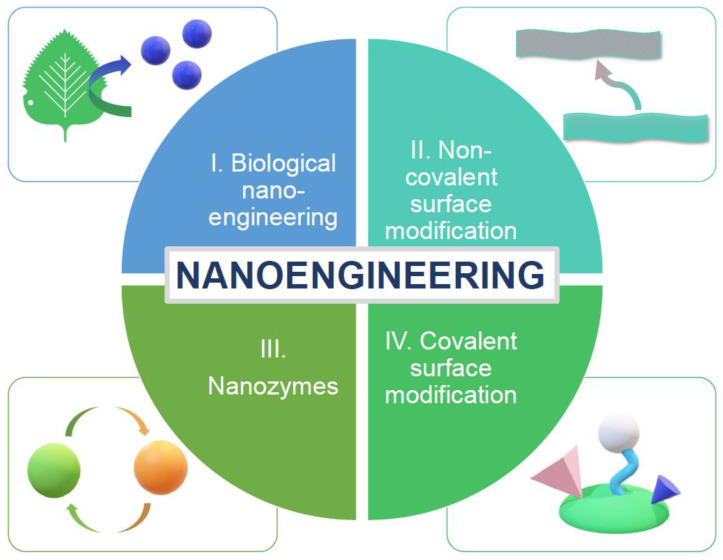
Different types of nanoengineering can be utilized to produce antioxidant nanostructures, such as (i) engineering directly from natural sources (e.g., nanoparticles’ extraction from plants), (ii) non-covalent surface modification of nanoantioxidants for optimization, (iii) synthesis of nanozymes, enzyme-mimicking nanoparticles, with inherent antioxidant activity, and (iv) covalent surface-modification, namely immobilization of a functional moiety into a support nanomaterial.

Nanoengineering has been adopted to optimize bio-medical antioxidant systems to improve stability, control release, enhance targeted administration, and overcome toxicity and biocompatibility issues [1,17]. Biomimetic nanoengineering can be realized by surface chemical modifications with a natural antioxidant, synthetic mimetic antioxidant, or by directly mimicking a natural antioxidant system (see Figure 13). Engineered artificial antioxidants can thus be divided into nanomaterials bearing antioxidant functionalities and nanomaterials with inherent antioxidant activity. Nanoengineering hybrid systems offer countless possibilities since they can (i) induce antioxidant properties to one initially inactive component, (ii) promote synergistic effects between the components of the hybrid system, and (iii) control the properties of support materials (e.g., minimize its toxicity). When designing new antioxidant materials, solubility, bioavailability, and safety should be considered [93].

Considering all the above-studied technologies and applications, the contribution of nanotechnology to producing advanced materials is undeniable. However, the extensive use of nanomaterials (NMs) due to the capacity of the nanoscale to be utilized in a variety of applications might introduce adverse effects and potentially threaten the environment and human health [57], [113,114]. Nanotoxicity can occur due to structural characteristics and synthetic pathways [115]. Minimizing nanotoxicity is, therefore, a parameter that must be considered when designing and engineering different nanotechnology-based materials [116]. Concepts such as the “Safe-by-Design” research philosophy facilitates the potential of a nanostructured material to be industrially produced [61].

### 5.1. Biological Nanoengineering

Besides traditional engineering methods, such as laser ablation, chemical vapor deposition, ion sputtering, chemical reduction, or sol-gel synthesis, antioxidant nanoparticles can also be synthesized via biological routes [117]. Metallic NPs can be synthesized biologically through bacteria, yeast, fungi, plants, or algae [118].

Nanoantioxidant Ag NPs have been extracted from *Clerodendrum phlomidis*, which belongs to the broader *Lamiaceae* family, and is well known as an antioxidant and anti-inflammatory in countries with tropical climates [119]. These green-synthesized Ag NPs show antioxidant activity compared to a ferulic acid standard of 910 AEAA > FA ≈ 710 AEAA (phosphomolybdate assay), 1.63 AU < FA ≈ 1.8 AU (ferric reducing power assay), IC50 = 55.86 μg/mL < IC50 = 202.2 μg/mL (superoxide anion radical scavenging), and IC50 = 9.12 μg/mL < IC50 = 182.8 μg/mL (DPPH method) [119]. Similarly, biocompatible nanoantioxidant carbon dots have been produced from tomato juice and evaluated as DPPH radical scavengers [120]. They present enhanced antioxidant capacity compared to hydrothermally obtained glutathione (GCD) [120].

**Figure 14:**
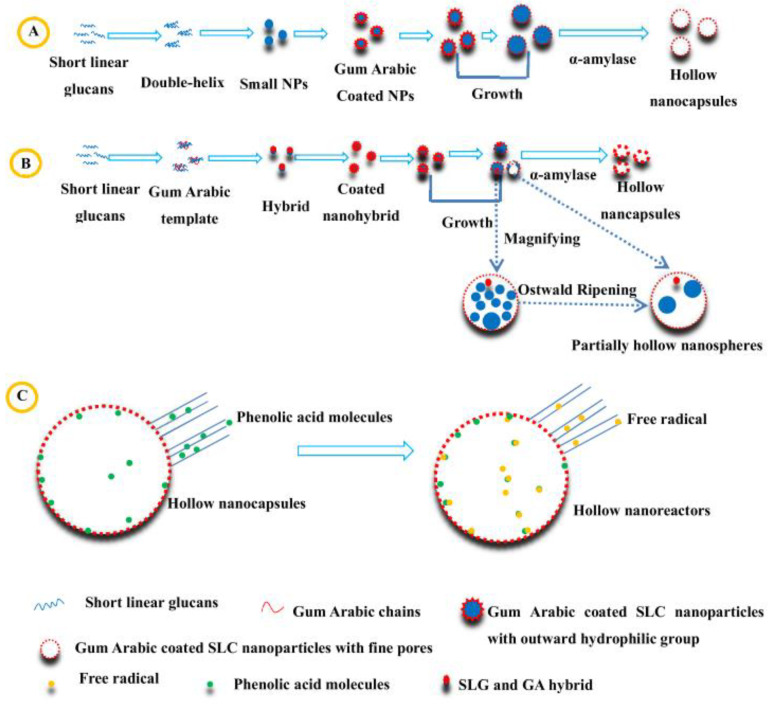
Schematic representation of the nanoengineering method and antioxidant mechanism of (**A**) hollow SLG@GA nanocapsules, and (**B**) in-situ SLG/GA hybrid and (**C**) phenolic acid/ free radicals reaction mechanism in the hollow nanoreactors. Reprinted (adapted) with permission from [40]. Copyright 2023 American Chemical Society.

**Figure 15:**
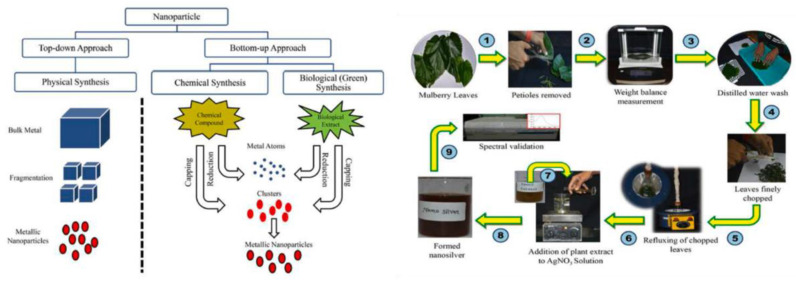
Schematic representation of (**left**) a top-down and bottom-up synthetic approach (**right**) of a phyto-synthetical Ag nanoparticles preparation. Reprinted (adapted) with permission from [121].

To enhance phenolic acid’s antioxidant activity and stability under harsh conditions, hollow short linear glucan (SLG)@gum arabic (GA) nanospheres and hollow in situ SLG/GA hybrid nanospheres have been engineered (See Figure 14). An α-amylase treatment and Ostwald ripening have removed the sacrificial starch nanoparticle templates. The encapsulated nanohybrids show an enhanced antioxidant activity towards DPPH radicals compared to their non-encapsulated counterparts (see Figure 12) [40]. Many more examples of nanoantioxidants engineered from natural sources, such as plant extraction, referred to in the bibliography, are listed in Table 2, along with the antioxidant mechanism and the utilized evaluation methods. Finally, Figure 15 depicts a top-down and bottom-up synthetic approach of a phyto-synthetical Ag nanoparticles preparation [121].

### 5.2. Nanoantioxidant Hybrids via Non-Covalent Modification

**Figure 16:**
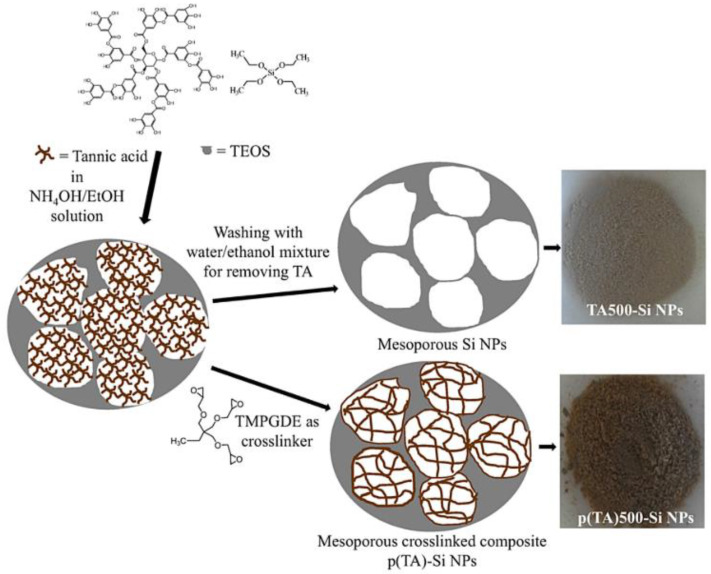
Schematic representation of an effective hybrid monodispersed nanoantioxidant p(TA)-Si NPs composite of tannic acid and silica nanoparticles via a modified Stöber method and one-pot synthesis. Reprinted (adapted) with permission from [27].

Sahiner et al. synthesized an effective hybrid monodispersed nanoantioxidant p(TA)-Si NPs (see Figure 16), a composite of tannic acid and silica nanoparticles via a modified Stöber method and a one-pot synthesis [27]. The physicochemical properties of the hybrid nanoantioxidants (such as particle size, dispersion profile, SSA, and pore characteristics) are controlled by the TA concentration of composite particles and the reaction time. The hybrids show significant RSC against ABTS radicals, while the synthetic procedure is a promising technique for the nanoengineering of tunable biochemical structures [27].

Moreover, Salazar et al. synthesized pure Lu_2_O_3_ NPs and doped them with Eu^3+^ Lu_2_O_3_ NPs by a sol-gel method [144]. An increase in the concentration of the materials also increased their antioxidant activity, which was evaluated by the ABTS method. The highest RSC is reached by Eu^3+^ doped Lu_2_O_3_ NPs (86%) [144]. Ti_3_C_2_ MXene nanosheets coated with hyaluronic acid-*graft*-dopamine (HA-DA)/polydopamine (PDA) have been created as non-enzymatic nanoantioxidants to neutralize RNS and ROS, including H_2_O_2_, O_2_^•−^, and ^•^OH, retaining the intracellular redox homeostasis, regulating oxidative stress, and eradicating bacteria to avoid infection [145].

In the same context, modifying carbon materials with substances exhibiting intrinsic biological activity concerning ROS enhances their antioxidant properties [48]. Polyethylene glycol (PEG) and polyvinyl pyrrolidone (PVP) capped nanoantioxidant CuO and ZnO NPs have been synthesized. Antioxidant activities are expressed in total flavonoid/phenolic content, total antioxidant capacity, total reducing power, and % inhibition of DPPH enhanced after capping [146,147].

**Figure 17:**
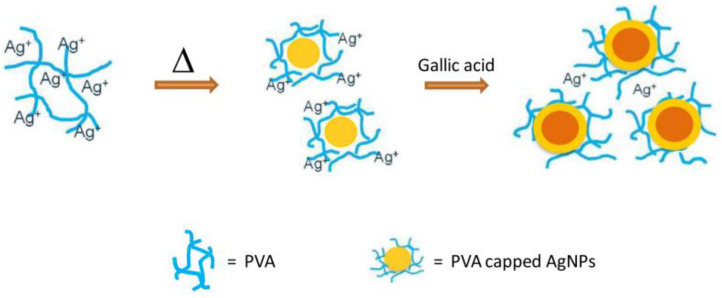
Schematic representation of the synthesis of PVA-AgNPs nanoantioxidant hybrids. Reprinted (adapted) with permission from [103].

Fe_2_O_3_ NPs, on the other side, hold a critical position in the field of biomedical technology. A simple one-pot hydrothermal precipitation method developed a Fe_2_O_3_/C hybrid nanoantioxidant with intrinsic superparamagnetic properties consisting of iron oxide NPS and encapsulated graphitic carbon [106]. To enhance the biocompatibility of the composite carbon layers formed by the carbonization of glucose were bound to the iron oxide surface through PEG. The RSC has been evaluated by monitoring the DPPH decay. Fe_2_O_3_/C NPs have an RSC of 89% [106]. Finally, to optimize the properties of bacterial cellulose (BC), a biopolymer composed of nanofibers, and to enhance its biomedical application capacity, silymarin SMN, which belongs to the group of flavonoids as well as zein, were joined together, creating spherical nanoparticles SMN-Zein [148]. The BC can absorb these nanoparticles. Tsai et al. prepared a new nanocomposite consisting of SMN-Zein/BC NPs. The ability of the SMN-Zein NPs and zein NPs to scavenge radicals was evaluated towards DPPH, ABTS, and hydroxyl radicals through UV–Vis spectroscopy, and was shown to be higher in the hybrid material in all cases [148]. Finally, Figure 17 schematically represents the synthesis of PVA–AgNPs nanoantioxidant hybrids [103], while Table 3 lists characteristic examples mentioned in the bibliography regarding non-covalent surface-modified nanoantioxidants and nanozymes.

### 5.3. Nano(en)zymes

**Figure 18:**
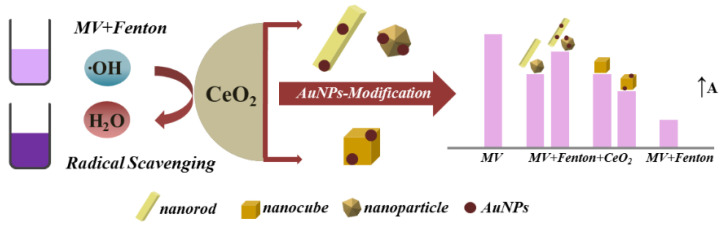
Schematic representation of the CeO_2_ surface-modification with AuNPs and their concentration-dependent antioxidant activity (A ↑). Reprinted (adapted) with permission from [101].

Nanoparticles that can behave and mimic the properties of enzymes can replace normal enzymes, also known as nanozymes [104]. An antioxidant nanohybrid LCNP, consisting of CeO_2_ NPs coated with levan polysaccharide (monomer form fructans), has been proposed [149]. The antioxidant activity was studied through the DPPH method. LCNPs present a concentration-dependent RSC of 85% against DPPH^●^ radical in pH = 7. In contrast, their RSC % reduces in pH = 4 [149]. Besides coating, a change in the morphology of CeO_2_ NPs can lead to a different antioxidant profile [101]. Au-modified CeO_2_ nanorods and nanoparticles show enhanced antioxidant activity, while their RSC decreases in the case of nanocubes (see Figure 18) [101]. Antioxidant sub-10 nm CNPs have been developed through an easy precipitation method at room temperature, while a post-synthetic surface silanization was utilized to increase dispersibility in biological media [55]. Glutathione peroxidase (GPx)-mimicking V_2_O_5_ nanowires have been engineered by a hydrothermal method, providing cytoprotection against harmful oxidative damage [100]. Similarly, engineered Fe_3_O_4_ NPs can mimic the activity of peroxidase and, thus, due to the Fenton reaction (Fe^2+^/Fe^3+^), can act against H_2_O_2_ [142]. Finally, Ragg et al. determined that, functionalized with dopamine, MoO_3_ NPs mimic the action of sulfite oxidase [143].

**Table 3:** List of (i) engineered nanoantioxidants developed through non-covalent surface modification and (ii) nanozymes.

	Nanoantioxidant	Target	Evaluation Methods/Antioxidant Efficiency *	Ref.
		*Non-covalent surface modification*		
1	Mesoporous poly-(Tannic Acid) (p(TA)-Si NPs)	ABTS^●+^	Total phenol content(TPC): GA equivalency_p(TA)1000eSi NPs_ = 14 ± 0.3 μg/mL TEAC: _p(TA)1000eSi NPs_ = 68± 6 mM Trolox equivalent g^−1^	[27]
2	PVA-Ag NPs, Poly(vinyl alcohol)	ABTS^●+^	ABTS^●+^:TAC values of ginger_Supplemental ginger capsule 3_ = 3.199 ± 0.025 mg gallic acid/g sample	[103]
3	Fe_2_O_3_/C	DPPH (^●^N)	^●^N (DPPH method): RSC _Fe2O3/C NPs_ = 89%, for 10 mg of Fe_2_O_3_/C in the solution	[106]
4	SMN-Zein/BC	DPPH ^●^N, ABTS^●+^, O_2_^●−^	^●^N (DPPH method): EC_50 ZeinNPs_ = 897.5 ± 21.4 μg/mL > EC_50 SMN-ZeinNPs_ = 38.5 ± 1.1 μg/mL ABTS^●+^: EC_50 ZeinNPs_ = 55.3 ± 2.5 μg/mL > EC_50 SMN-ZeinNPs_ = 38.5 ± 1.1 μg/mL O_2_^●−^: EC_50 ZeinNPs_ = 3213.5 ± 165.7 μg/mL > EC_50 SMN-ZeinNPs_ = 214.7 ± 6.9 μg/ml	[148]
5	Au/CeO_2_	^●^OH	Concentration-dependant improvement/inhibition of antioxidant capacity in hybrids vs. CeO_2_.	[101]
6	^d^ Fe_3_O_4_ NPs	H_2_O_2_	Peroxidase-like activity	[150]
7	Lu_2_O_3_ NPs-doped with Eu^3+^	ABTS^●+^	ABTS^●+^: RSC = 86%	[144]
8	CuO-PEG, CuO-PVP	DPPH (^●^N)	TAC_CuO-PVP_ = 32.44 ± 0.1 (μg AAE/mg) > TAC _CuO-PEG_ = 27.42 ± 0.24 (μg AAE/mg) > TAC_CuO_ = 18.94 ± 0.57 (μg AAE/mg) TRP_CuO-PVP_ = 17.38 ± 0.15 (μg AAE/mg) > TRP _CuO-PEG_ = 16.64 ± 0.2 (μg AAE/mg) > TRP_CuO_ = 7.10 ± 0.3 (μg AAE/mg) ^●^N (DPPH method): RSC_CuO-PEG_ = 34.14% > RSC _CuO-PVP_ = 28.36% > RSC_CuO_ 13.79%	[147]
9	ZnO-PEG, ZnO-PVP	DPPH (^●^N)	TAC_ZnO-PEG_ = 22.8 ± 1.55 (μg AAE/mg) > TAC_ZnO-PVP_ = 19.1 ± 1.64 (μg AAE/mg) > TAC_ZnO_ = 13.1 ± 1.11 (μg AAE/mg) TRP_ZnO-PVP_ = 15.1 ± 1.65 (μg AAE/mg) > TRP _ZnO-PEG_ = 13.5 ± 1.13 (μg AAE/mg) > TRP _ZnO_ = 6.64 ± 0.05 (μg AAE/mg) ^●^N (DPPH method): RSC _ZnO-PVP_ = 13.75% > RSC_ZnO-PEG_ = 13.66% > RSC_ZnO_ = 9.66%	[146]
10	Chi-SiO_2_, Chi-CMC-SiO_2_	DPPH (^●^N)	^●^N (DPPH method): RSC_Chi-CMC-SiO2_ = 1.5 RSC_ChiSiO2_	[151]
11	Chi-Ppy, chi-PPy-PTDA	DPPH (^●^N)	^●^N (DPPH method): max RSC = 86%	[152]
12	^b,c^ MoS_2_@TiO_2_	ROS	Strong bionic bi-enzyme activity	[153]
13	^c^ CDs-CeO_2_ nanocomposites	H_2_O_2_	Enzyme-like activity	[154]
14	^a^ Cs-FeO	DPPH (^●^N), H_2_O_2_	^●^N (DPPH method): maxRSC_CS-FeO_ ≈ 93% > RSC_FeO_ ≈ 83% H_2_O_2_: maxRSC_CS-FeO_ ≈ 82% > RSC_FeO_ ≈ 72%	[43]
15	Pd-RGO-ZnO	DPPH (^●^N), ^●^NO	^●^N (DPPH method): max RSC_Pd-RGO-ZnONPs_ = 58.0% > RSC_RGO-ZnONPs_ = 45.2% > RSC_RGO_ = 27.5%/^●^NO: Max RSC_Pd-RGO-ZnONPs_= 48.6% > RSC_RGO-ZnONPs_ = 39.3% > RSC_RGO_ = 27.8%	[155]
16	Rpda NPs, dextran/chitosan	DPPH (^●^N), ABTS^●+^	^●^N (DPPH method): max RSC_rPDA NPs_ = 85% ABTS^●+^: max RSC_rPDA NPs_ = 90%	[156]
17	L-PDNPs	^●^OH	Cell protection from/decreasing ROS-induced damages/alteration.	[157]
18	^a^ CUR-AuNPs AuNPs and co-functionalization with Curcuma pseudomontana isolated curcumin (CUR)	DPPH (^●^N), H_2_O_2_, ^●^NO	^●^N (DPPH): max RSC_VitaminC_ = 89.6% > max RSC_CUR-AuNPs_ = 85.2% > max RSC_CUR_ = 84.2%, H_2_O_2_: max RSC_Vitamin C_ = 84.8% > RSC_CUR-AuNPs_ = 83.2% > RSC_CUR_ = 76.5%, (RP): max RSC_VitaminC_ = 91.4% > RSC_CUR-AuNPs_ = 87.9% > RSC_CUR_ = 82.3%, ^●^NO: max RSC_Vitamin C_ = 84.8% > RSC_CUR-AuNPs_ = 84.5% > RSC_CUR_ = 79.5%	[158]
19	^a^ Quercetin–linseed oil co-loaded lipid carrier (NLCS)	DPPH (^●^N)	^●^N (DPPH): max RSC_Quercetin NLCS 3_ ≈ 77%	[159]
20	^a^ Turmenic extract encapsulated in NLC, T-NLC	DPPH (^●^N)	^●^N (DPPH): max RSC_T-NLC_ ≈ 45% > RSC _turmeric extract_ ≈ 40%	[160]
21	Zein-pectin NPs loaded with curcumin	DPPH (^●^N), ABTS^+●^	^●^N (DPPH): SC_50 Curcumin_ ≈ 17.5 μg/mL > SC_50 Zein-pectin NPs loaded with curcumin_ ≈ 14.7 μg/mL > SC_50 AA_ ≈ 5.5 μg/mL ABTS^+●^: TEAC _Zein-pectin NPs loaded with curcumin_≈14.3 mg > TEAC _Curcumin_ ≈0.8 mg > TEAC _Zein-pectin NPs_ ≈ 0.04 mg	[161]
22	Ti_3_C_2_ MXene nanosheets	RNS, ROS (H_2_O_2_, O_2_^•–^, and ^•^OH)	Scavenging excessive RNS and ROS	[145]
		*Nanozymes*		
23	CNPs (Cerium nanoparticles)	^●^OH	Enzyme-like activity	[55]
24	LCNPs	DPPH (^●^N)	^●^N (DPPH): max RSC _LCNPs_ = 85%	[149]
25	V_2_O_5_	NADPH	Enzyme-like activity	[104]

^a^ Hydrogen Atom Transfer (HAT) proposed mechanism, ^b^ SOD-mimetic activity, ^c^ CAT-mimetic activity, ^d^ GPx-mimetic activity. ∗ Antioxidant efficiency is listed as and where referred to by authors.

### 5.4. Surface Chemical Modification of Nanomaterials by Grafting Natural Antioxidants or Functional Components to Produce Hybrid Nanoantioxidants

The chemical modification/functionalization of a nanomaterial surface represents a growing research field, and it is a widely used nanoengineering concept in controlling different parameters of chemical systems. The unique properties provided by the nanoscale, combined with functionalities, lead to advanced structures for specific applications. Thus, surface functionalization processes are used to develop heterogenized antioxidant nanostructures targeting (i) optimization of their inherent efficiency as antioxidants (e.g., radical scavenging capacity (RSC)) and redox-regulated performance, (ii) induction or enhancement of different properties (e.g., antibacterial), (iii) control of their potential toxicity and adverse effects towards humans and the eco-system, and (iv) facilitation of their synthetic procedure to increase their potential to be industrially produced [9].

Functionalization can have different approaches; the first would be immobilizing a functional moiety onto an inert inorganic matrix to produce a nanohybrid that preserves the properties of the functional molecule (e.g., antioxidant activity). At the same time, the support provides a non-functional role (e.g., by offering stability) or a synergistic effect (e.g., enhancement of the antioxidant capacity). Another approach of surface functionalization can be found in grafting a non-functional moiety onto a reactive support material to produce a nanohybrid where a surface-initialized phenomenon can (i) induce antioxidant properties to one initially inactive component of the hybrid system [15], (ii) promote synergistic effect between the components of the hybrid system [25], (iii) control the properties of the support material (e.g., minimizing its toxicity), or (iv) overcome inherent structural disadvantages (e.g., aggregation). Functional groups, such as –COOH, –NH_2_, –OH, and –SH, can interact with bioactive ligands and be incorporated into ligand addition, ligand exchange, or encapsulation processes [9].

Various functionalization protocols have been utilized to develop diverse advanced nanoantioxidant structures. Among these are hybrid core-shell structures from a combination of natural antioxidants with the properties of minerals, created by immobilization onto nanoparticles. The described systems have been chosen based on their evaluation as antioxidants, to compare the efficacy of different hybrid nanoantioxidant systems. Table 4 lists several examples mentioned in the bibliography regarding covalent surface-modified nanoantioxidant hybrids, while some characteristic studies are described here.

Caffeic acid (CA) has been immobilized onto plasma-treated ZnO nanoparticles to create a hybrid ZnO@CA nanoantioxidant through the covalent bond formation between the carboxyl group of CA and Zn ions [25]. The radical scavenging capacity (RSC) of the hybrid ZnO@CA (73.68%) towards the cationic ABTS radical was evaluated by a decolorization assay and is concentration-dependent and lower than the RSC of CA (93.25%), possibly due to steric repulsive forces between nanometer-sized ZnO and the ABTS radicals [25]. The antioxidant activity is provided by the CA and not the initially inactive nanoparticles, which is also corroborated by the work of Fan et al. [22]. In this study, hybrid bovine serum albumin (BSA) and CA have been developed by radical-induced grafting conjugation, to be used as an emulsifier to stabilize resveratrol-loaded zein nanoparticles. The fluorescent DPPH assay evaluated the antioxidant capacity of the hybrid BSA-CA, ferric reducing power, and ORAC method, and indicated that BSA-CA (89.7% at 0.4 mg/mL) shows clearly better antioxidant activity than BSA (9.0%), but not better than CA (91.9% at 0.4 mg/mL) [22]. Resveratrol alone has a lower antioxidant activity than the zein-BSA and zein-BSA-CA nanoparticles [22]. CA has also been immobilized onto the surface of MSNs in a two-step surface modification treatment. First, amino-functionalized core-shell silica nanospheres were created with the conjugation of the caffeic acid onto the nanospheres to create the hybrid material (ACSSNs-CA). The functionalization of caffeic acid into the ACSSNs surface was achieved by the reaction of the -COOH group from the CA and the -NH_2_ group of the ACSSNs, with the help of coupling reagents EDC/NHS [19]. The RSC, evaluated by the DPPH method, follows the SPLET mechanism. In this case, increasing the CA concentration in the hybrid materials can enhance the overall RSC of the material [19].

**Figure 19:**
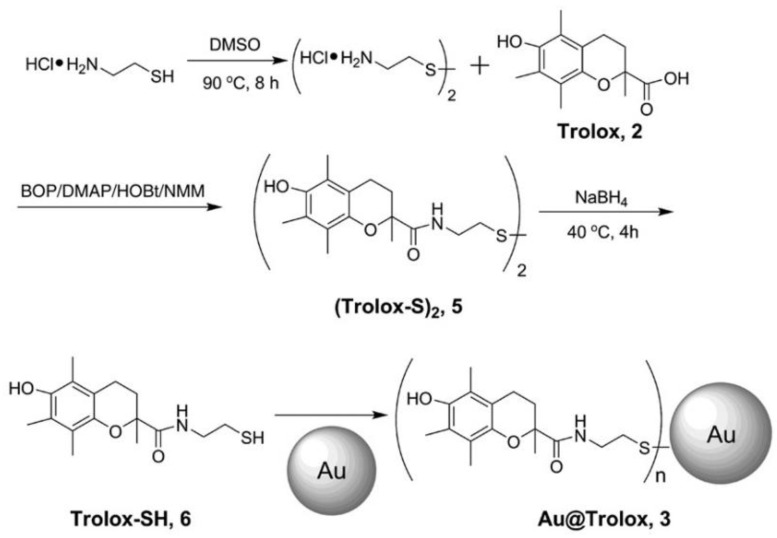
Schematic depiction of the synthetic procedure used to prepare Au@Trolox nanoantioxidant. Reprinted (adapted) with permission from [38].

Trolox, a water-soluble analog of vitamin E (or tocopherol)*,* has been functionalized onto Au nanoparticles to produce a hybrid Au@Trolox nanoantioxidant system with improved properties (see Figure 19) [38]. A self-assembly process was held to achieve the functionalization of the thiol ligands of Trolox onto the Au NPs. The RSC of the hybrid material was assessed by the DPPH assay and in with a stopped-flow electron spin resonance (ESR) technique. Unlike the CA hybrid nanoantioxidants, mentioned above, the rate constant of Au@Trolox is eight times higher than that of the unfunctionalized Trolox. Moreover, in this case, the antioxidant activity is not affected by the concentration of the immobilized antioxidant molecule [38]. Another study presents hybrid Trolox nanoantioxidants functionalized onto Se nanoparticles by self-assembly [37]. The hybrid nanoantioxidant Se@Trolox can reduce the formation of ABTS free radicals, and its RSC is time-dependent. Again, the hybrid Se@Trolox shows an enhanced antioxidant capacity compared to the unfunctionalized Trolox [37]. One more work indicates the development of hybrid nanoantioxidant structures of Trolox functionalized onto biocompatible pegylated gold nanoparticles to reduce oxidative stress and neurotoxicity [162]. The antioxidant activity of the hybrid Au@PEG (Au@Trolox) was evaluated using the DPPH method, presenting an enhanced RSC compared to the unfunctionalized Trolox, and the simple mixture of Au@PEG and Trolox, indicating that the nanoengineering enhances the antioxidant activity due to the π−π* stacking, formed between the adjacent phenolic moiety that exists on the surface Au NPs [162].

A hybrid material, Au@PEG3SA, originated initially from the coating of the PEG on the gold nanoparticles through self-assembly and then the functionalization of the salvianic acid (SA) on the surface of the PEG-coated AuNPs [38]. The RSC, evaluated by the decay of DPPH and kinetic analysis, increases in the hybrid compared to its monomer SA [38]. Massaro et al. prepared a double hybrid nanoantioxidant system by selectively grafting Trolox on the external surface of halloysite nanotubes (HNTs) and concurrently loading quercetin into the inner lumen to create a bi-functional nanoantioxidant [163]. The evaluated RSC towards peroxyl radicals of the double nanohybrid HNT–Trolox/Que was 35% higher than the mono-functional analogs HNT–Trolox and HNT/Que. The synergistic effect of the distinct antioxidants, confirmed by the RSC towards DPPH radicals, was described as a rapid reaction of the external Trolox regenerated by the released quercetin [163].

**Figure 20:**
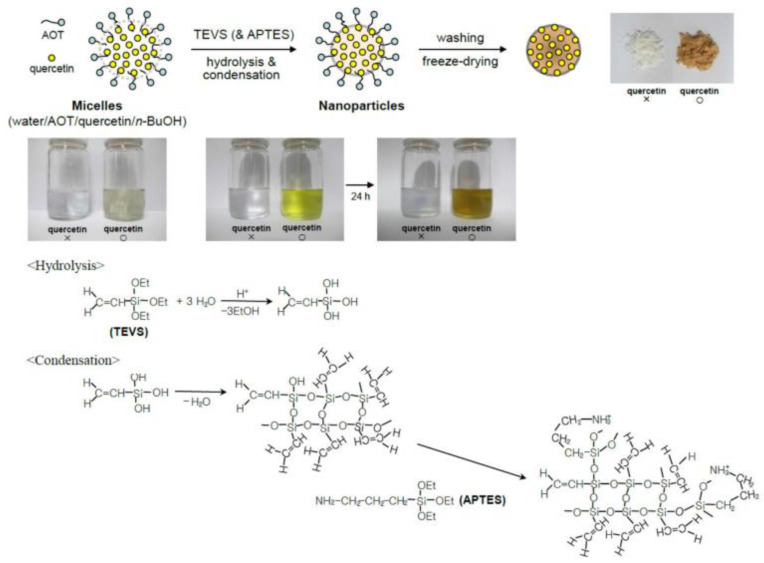
Schematic representation of the preparation of quercetin-loaded silica nanoantioxidants. Reprinted (adapted) with permission from [165].

Quercetin: Hybrid nanomaterials PLGA-Que with different loadings were created, consisting of poly-lactide-co-glycalic acid (PLGA) NPs and quercetin, aiming mainly at improving the aqueous solubility. The antioxidant activity influenced the amount of PLGA, reaching a max RSC = 80% of DPPH radicals in a medium concentration [34]. A mixture of quercetin and biapigenin was isolated from *H. Perforatum* and encapsulated into PCL NPs utilizing a solvent displacement method, with the optimal material having the proportion of PCL: compounds of 1:0.1 [164]. The antioxidant activity of PCL-Oue NPs was evaluated through DPPH, superoxide radical scavenging activity, and iron (II) chelating activity. Encapsulation did not alter the DPPH RSC of the system. However, the superoxide radical scavenging ability of the hybrid could not be detected, while the iron (II) chelating activity was significantly higher than quercetin–diapigenin [164]. Additionally, Ag–Se nanoparticle support has been combined with the natural antioxidants quercetin (flavonol) and GA (phenol) to create a nanohybrid antioxidant material [33]. Three methods, DPPH, ABTS, and MTT (method not described in this work-mainly used for an anticancer capacity evaluation), were used to evaluate the antioxidant activity of the bimetallic (Ag–Se) nanoparticles functionalized with natural antioxidants [33]. The antioxidant activity of the hybrid material is determined by GA, which exists in a small percentage on the surface, and quercetin, which is also contained in a more significant percentage [33]. Phenols and flavonols components can scavenge free radicals by donating a hydrogen atom or by scavenging singlet oxygen. The results from the three methods for the (Ag–Se) nanoparticles by quercetin and GA were at 50 μg/mL, ABTS:62.54%, DPPH: 59%, and MTT: 61%; thus, the hybrid material can be considered a good antioxidant. The antioxidant activity of quercetin-loaded silica nanoantioxidants is depicted in Figure 20 [165].

**Figure 21:**
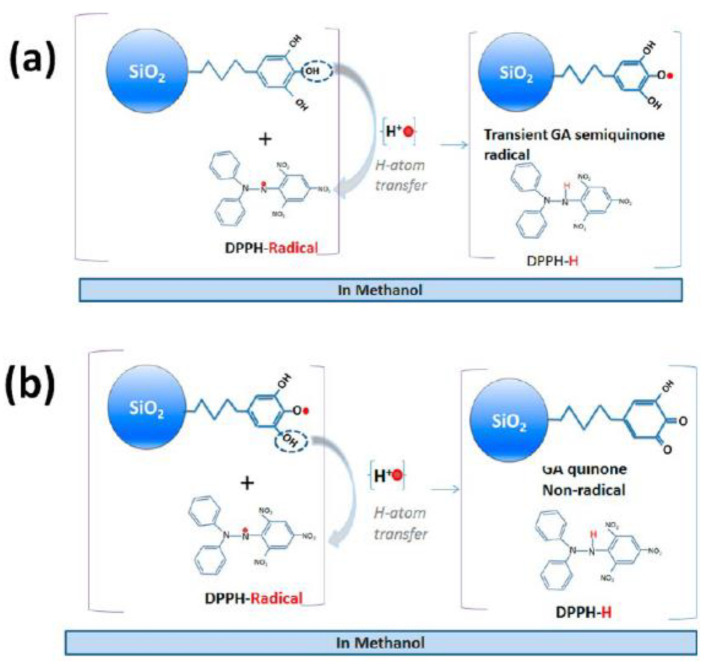
Schematic representation of the Hydrogen-Atom Transfer [HAT] from (**a**) the GA molecule forming to a transient GA radical, and (**b**) a GA semiquinone forming a nonradical GA quinone. Reprinted (adapted) with permission from [88]. Copyright 2023 American Chemical Society.

Among the natural and synthetic antioxidants used to create nanohybrids, Gallic Acid (GA) plays an important role. Its unique properties reflect its capacity to be utilized in various applications. In this work, the magnetite IONP nano surface was functionalized with GA through in situ and post-synthesis methods to improve the properties of the nanoparticles [23]. Three types of IONP-GA hybrid materials were created with different sizes, and their RSC was evaluated through the DPPH method. The antioxidant activity was enhanced in all three hybrids compared to the unfunctionalized support [23]. In addition, our group created covalently grafted GA molecules onto the nanosilica surface (see Figure 18) [88]. Two spectroscopic methods were used to evaluate the antioxidant activity of the SiO_2_-GA nanohybrids: Electron paramagnetic resonance (EPR) to directly detect the formed radicals, and UV–Vis to evaluate the decay of the DPPH radical [88]. The results stated that the SiO_2_-GA nanoantioxidants scavenge the DPPH radical through the HAT mechanism (Figure 21). Kinetic analysis results show that the reaction between SiO_2_-GA and the DPPH radical includes multiple phases, an n_fast_ (t_1/2_ < 1 min), where the HAT mechanism occurs, and an n_slow_, where radical–radical reactions take place [88]. SiO_2_ [90]-GA NPs can scavenge 4.1 (±0.2) µM of DPPH radicals, corresponding to n_fast_ = 2.1 ± 0.2 [88].

**Figure 22:**
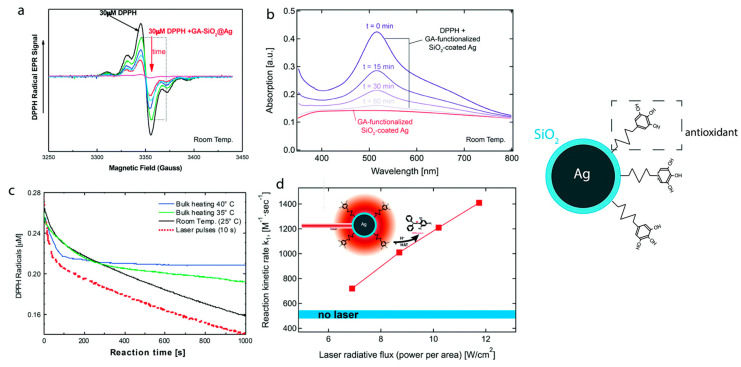
DPPH assay was used to evaluate the antioxidant activity of plasmonic Ag NPs grafted with gallic acid. Kinetic studies indicate a rapid phase at the beginning that determines the RSC towards DPPH^●^ radicals [166]. During the fast phase, the SiO_2_@Ag@GA scavenges the DPPH^●^ radical due to the 2-electron/2-proton reactions per GA molecule. After the grafting, the bond dissociation enthalpy of the GA-OH bond decreases by 2 kcal/mol. (**a**) and (**b**), EPR and UV–Vis spectra of SiO_2_@Ag@GA, (**c**) and (**d**) DPPH decay kinetics. Reprinted (adapted) with permission from [166]. Copyright 2023 American Chemical Society.

Additionally, Sotiriou et al. synthesized hybrid plasmonic (exhibited a plasmonic effect at near-IR wavelengths) NPS through the functionalization of GA into Ag plasmonic NPs with an outer coating of silica (see Figure 7 and Figure 22) [166]. The DPPH assay was used to evaluate the antioxidant activity of those nanoparticles. Kinetic studies indicate a rapid phase at the beginning that determines the RSC towards DPPH^●^ radicals [166]. During the fast phase, the SiO_2_@Ag@GA scavenges the DPPH^●^ radical due to the 2-electron/2-proton reactions per GA molecule. After the grafting, the bond dissociation enthalpy of the GA-OH bond decreases by 2 kcal/mol [166].

Poly(lactic-co-glycolic acid) (PLGA) NPs coated or not with polysorbate 80 (PS80) containing GA have been prepared and evaluated as antioxidants by a colorimetric measure of the radical cation ABTS^•+^. The NP-PLGA-GA shows better antioxidant activity than NP-PLGA/PS80-GA, but worse than GA [167]. Similarly, Lee et al. synthesized a hybrid material consisting of ZnO NPs as an inorganic matrix and GA as an organic functionalized moiety, covalently bonded together to form the hybrid ZnO@GA NPs (2.89 GA molecules per particle) [168]. The ABTS assay studied the RSC of the hybrid ZnO@GA NPs and compared it with GA. ZnO@GA NPs exhibit an RSC% equal to 69.71 at the highest concentration (100 μΜ), while GA shows an RSC% equal to 93.25 at 100 μΜ. The lower ability of the hybrid ZnO@GA NPs vs. GA to scavenge ABTS^●+^ radicals is assigned to the steric repulsion between ZnO@GA NPs and ABTS^●+^ [168].

As a way of utilizing the inherent antioxidant activity and concurrently overcoming the limitations of natural polyphenols, a study describes the synthesis of hybrid nanoantioxidant materials using starch nanoparticles (SNPs) and four different polyphenols: (+)- catechin (C), (-)-epicatechin (EC), (-)-epigallocatechin-3-gallate (EGCG), and proanthocyanidins (PAG) [111]. RSC towards DPPH radicals combined with kinetic analysis indicated the improved properties of the immobilized polyphenols [111]. This finding could lead to developing applications based on SNPs as a carrier of bioactive compounds [111].

Two natural antioxidants, caffeic acid (CA) and rutin, were covalently grafted on mesoporous *silica* nanoparticles to create two nanohybrids antioxidants, MSN-CAF, and MSN-RUT, respectively [35]. The hybrids MSN-CAF and MSN-RUT were studied for their ability to scavenge radicals through the ORAC method, and the results show that MSN-RUT has a much higher antioxidant activity than MSN-CAF [35].

Interestingly, it is presented that natural rutin shows a slightly greater antioxidant effect than natural CA. The MSN-CAF hybrid material shows little antioxidant activity, and less than natural caffeic acid [35]. Das et al. took a flavone, 3-hydroxy-4′-methoxyflavone, and immobilized it on the amino-modified surface of mesoporous silica nanoparticles (MSN-APTES) [36]. Silica nanoparticles do not fluoresce, but if they can fluoresce when immobilized with a fluorescent molecule, this could be an advantage for the nanotechnology industry [36]. FMFS NPs developed by Das et al. were tested for their ability to scavenge radicals by the ferric-reducing power assay [36]. The RSC of the hybrid FMFS NPs was compared with those of GA; the FMFS NPs appear to exhibit remarkable antioxidant activity at 55.6 ± 0.06 mM of GA equivalent (GAE)^g−1^ [36].

Silica is, moreover, one of the materials that can be used to create Pickering emulsions because its surface is easily modified. If pigments are combined with inorganic matrices or natural polyphenols, hybrid materials combine their monomers’ properties. The hybrid antioxidant system exhibits pH-depended scavenging activity towards singlet oxygen and improves the oxidative stability in cosmetic Pickering emulsions [27]. In this context, a bifunctional pigment-antioxidant nanomaterial based on carminic acid covalently linked onto the amino-functionalized core-shell type of silica nanostructure (dense silica core with a mesoporous silica shell) was created [28].

Morin (2′,3,4′,5,7-pentahydroxyflavone), a phenolic compound found in vegetables, was immobilized onto MSNs, to produce the hybrid nanoantioxidant MSN-morin [32]. The antioxidant capacity of the hybrid material MSN-morin was examined towards hydroxyl radical (HO^●^) scavenging by EPR spectroscopy and singlet oxygen (^1^O_2_) monitoring its time-resolved phosphorescence [32]. The activity of the hybrid nanoantioxidant AMSNPs-MOR increases by 57% compared to the unfunctionalized morin molecule. The singlet oxygen assay results show that the k_T_ of AMSNPs-MOR is equal to 4.5 × 10^7^ M^−1^s^−1^, compared to the k_T_ of morin, which is equal to 1.3 × 10^8^ M^−1^s^−1^ [32].

Cellulose fiber was immobilized onto gold NPs (code-named UBK-AuNPs) [169]. The RSC of the nanoantioxidant was evaluated by a DPPH assay and UV–Vis Spectroscopy. The hybrid UBK-AuNPs has a scavenging rate of 86.05% ± 0.009 in the light and 77.86% ± 0.006 in the dark, higher than that of UBK at 47.7% [169]. Similarly, 3,6-dihydroxyflavone, lutein, and selenium methyl selenocysteine hybrids immobilized onto Au NPs have been developed and evaluated as antioxidants, using DPPH, ^●^OH, H_2_O_2,_ and ^●^NO radical scavenging assays, and compared to ascorbic acid (standard) [105]. Among the various combinations studied, the triplet hybrid 3,6-dihydroxyflavone, lutein, and selenium methyl selenocysteine (1:1:1) exhibited enhancement in the target activity at the same concentrations. Synthesized gold nanoparticle-embedded 3,6-dihydroxyflavone further enhanced the antioxidant activity [105].

**Figure 23:**
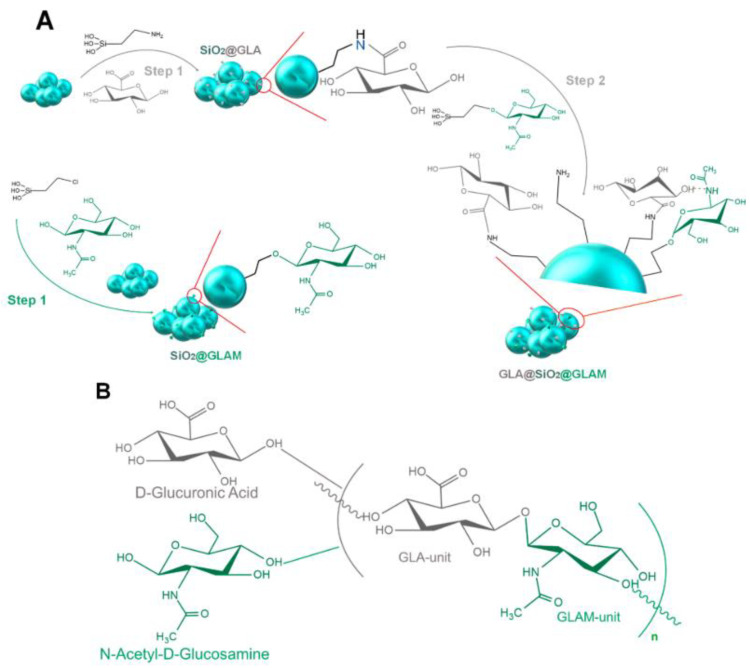
Schematic depiction of (**A**) the synthesis of the nanoantioxidant hybrids of covalent attached hyaluronic acid components on the surface of nanosilica, and (**B**) the structural units of hyaluronic acid, namely D-Glucuronic Acid, and N-Acetyl-D-Glucosamine. Reprinted (adapted) with permission from [15]. Copyright 2023 American Chemical Society.

From our side, in our recent work, we developed a new family of hybrid nanoantioxidants (see Figure 23) based on hyaluronic acid (HyA) components [D-glucuronic acid (GLA) and N- acetyl-D-glucosamine (GLAM)] covalently grafted on SiO_2_ NPs, SiO_2_@GLA, SiO_2_@GLAM, and GLA@SiO_2_@GLAM with a molar ration of [GLA: GLAM] [2:1], and [3:1] [15]. RSC has been evaluated by monitoring the DPPH decay through UV–Vis spectroscopy. The hybrid nanoantioxidants enable significant HAT activity versus DPPH radicals, while the unfunctionalized HyA counterparts are practically inactive [15]. The doubly grafted {GLA@SiO_2_@GLAM} nanohybrid with a molar ration of [GLA: GLAM] [2:1] shows the HAT mechanism [15]. The {GLA@SiO_2_@GLAM} with a molar ration of [GLA: GLAM] [3:1] follows, presents n_fast_ = 1.1 and Ea = 46.6 ± 1 kJ/mol [15], and the highest antioxidant activity (n_fast_ = 1.1, Ea = 42.2 ± 1 kJ/mol), due to local H-bonding phenomena between the SiO_2_ matrix, GLA, and GLAM that decrease the activation barrier.

**Figure 24:**
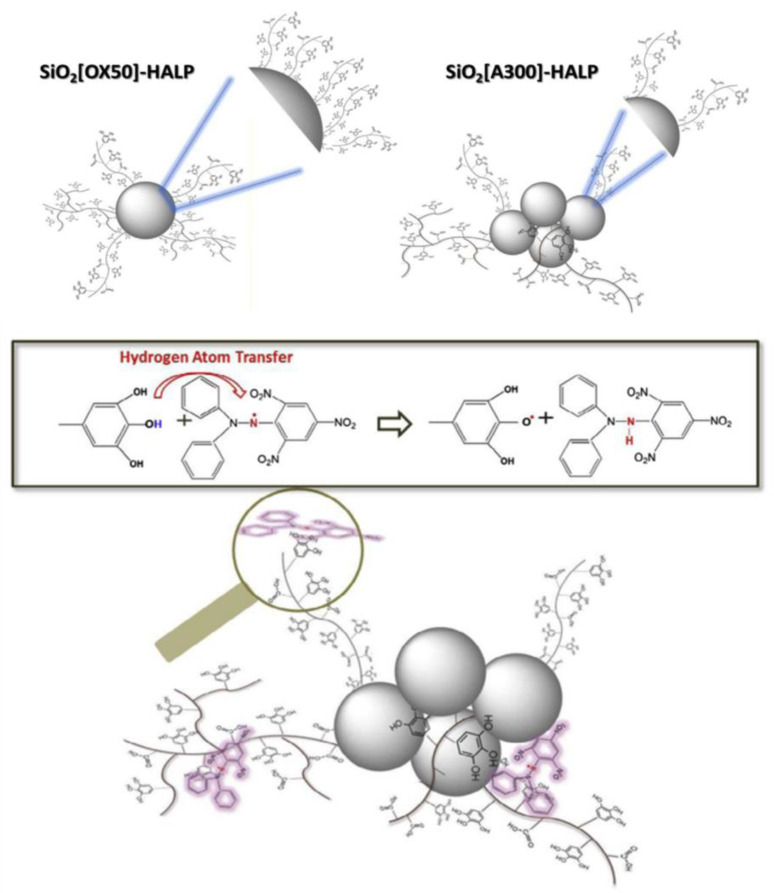
Schematic depiction of the antioxidant activity of engineered SiO_2_–HALP nanohybrids evaluated by assessing their kinetics for HAT towards DPPH radicals. Reprinted (adapted) with permission from [29].

Our group created nanohybrid materials by covalent grafting a polyphenolic polymer (Humic Acid Like Polycondensate (HALP)) on SiO_2_ NPs of different SSA (see Figure 24) [29]. The antioxidant activity of the SiO_2_–HALP nanohybrids was evaluated by assessing their kinetics for HAT towards DPPH radicals. It was shown that surface-grafted HALPs perform 300% better HAT than non-grafted HALP in solution [29]. Moreover, the HAT performance can be optimized by controlling the particle type and grafting loading [29].

Marulasiddeshwara et al. synthesized, utilizing green methods, a hybrid material that consists of natural lignin-capped Ag NPs [170]. The synthesis of the hybrid material was carried out, with the help of various coupling reagents, to create the hybrid LCSN [170]. The results of the RSC, evaluated by a DPPH assay of the hybrid material LCSN, showed that the material has an RSC = 70%, while expressed in IC_50_ for scavenging on DPPH, IC_50_ = 3360 μg/mL [170]. An antioxidant nanohybrid HNT/AH_2_ by a selective loading of vitamin C (ascorbic acid, AH_2_) immobilized onto halloysite nanotubes (HNT) has been prepared to stabilize vitamin C and tested for its RSC against DPPH^•^ and peroxyl radicals [171]. The nanohybrid HNT/AH_2_ showed higher RSC than the unfunctionalized ascorbic acid, namely 131% in acetonitrile and 290% in an aqueous solution [171]. Antônio et al. proceeded to prepare a material consisting of poly(lactic) acid (PLA) NPs, and ursolic acid (UA), PLA-UA NPs [100]. The synthesis of the PLA-UA NPs was carried out through the emulsification-solvent evaporation technique. The antioxidant activity of both PLA-UA NPs and free UA was evaluated through the HOCl (Hypochlorous acid scavenging capacity) method. PLA-UA NPs have a similar antioxidant effect to the free UA [100].

Nayak et al. created nanomaterials by joining Ag NPs derived from Hibiscus rosa-sinensis (HRS) with chitosan (HRS-Ag NPs-Chi) [41]. The antioxidant activity of the material was evaluated by DPPH, H_2_O_2_, FRAP, and nitric oxide scavenging assays. The nanoformulations showed higher antioxidant activity than their base counterparts. Thus, combined with their anti-cancer efficacy, the prepared formulations can be studied for breast cancer therapy [41]. A hybrid nanomaterial consisting of amino-functionalized MSNs and immobilized rosmarinic acid (RA) has been prepared [172]. These kinds of nanoformulations are essential to the biomedical field, as they enhance the administration of drugs. The antioxidant activity of the hybrid nanoantioxidant AMSN-RA was evaluated using a DPPH scavenging assay and shown to be concentration-dependent; as the concentration increases, so does the RSC%. At all concentrations, RA (RSC%) > AMSN-RA (RCS%), except at the concentration of 10 μg/mL [172]. In a recent study, superparamagnetic iron oxide nanoparticles (SPION) have been post-synthetically capped with phenolic compounds (GA, Trolox and nordihydroguaiaretic acid), applying different degrees of purification [58]. The nanohybrids act as antioxidants, trapping alkyl peroxyl (ROO^•^) radicals, an effect that strongly depends on the degree of their purification [58].

**Table 4:** List of engineered hybrid nanoantioxidants developed through grafting of natural antioxidants or functional components.

	Nanoantioxidant	Target	Evaluation Methods/Antioxidant Efficiency *	Ref.
1	^a^ Gallic acid at Silica NPs (SiO_2_@GA)	DPPH (^●^N)	^●^N (DPPH method): n_fast_ = 2.1 ± 0.2	[88]
2	^f^ Mesoporous SiO_2_ NPs (MSN) functionalized with morin AMSNPs-MOR	^●^OH, ^1^O_2_	^●^OH: RSC _AMSNPs-MOR_ = 57% higher than morin, ^1^O_2_:k_TAMSNPs-MOR_ = 4.5 × 10^7^ M^−1^s^−1^ < k_TMOR_ = 1.3 × 10^8^ M^−1^s^−1^	[32]
3	MSNs (MSN-CAF), rutin (MSN-RUT), where CAF = caffeic acid, and RUT = rutin	ROO^●^	ORAC_MSN-RUT_ = 7.32 ± 1.93 μmol/L TE < ORAC_RUT_ = 10.92 ± 1.73 μmol/L TE	[35]
4	(Cellulose fiber)-Au NPS	DPPH (^●^N)	^●^N (DPPH method): max RSC_UBK-AuNPs_ = 86.05% ± 0.009% > RSC_UBK_ = 47.7%	[169]
5	Au@PEG3SA (salvianic acid)	DPPH (^●^N)	^●^N (DPPH method): k_obs Au@PEG3SA_ = 65.3 ± 1.65 M^−1^ s^−1^ > k_obsSA_ = 7.13 ± 0.55 M^−1^ s^−1^	[173]
6	Au@Trolox	DPPH(^●^N)	^●^N (DPPH method): SR_Au@Trolox_ = 8 SR_Trolox_	[38]
7	^a^ Au NPs embedded 3,6 dihydroxyflavone, lutein, and selenium methyl selenocysteine	^●^N, ^●^OH, H_2_O_2_, ^●^NO	^●^N (DPPH method): RSC _AA_ = 96.28% > RSC _Au-triplet NPs_ = 87.13% RSC _3,6 dihydroxyflavone, lutein, and selenium methyl selenocysteine_ = 72.89% > RSC _Au-3,6 dihydroxyflavone_ = 72.04% > RSC _lutein_ = 65.79% > RSC _3,6 dihydroxyflavone_ = 65.79% > RSC _selenium methyl selenocysteine_ = 43.85% ^●^OH:RSC _AA_ = 96.18% > RSC _Au-triplet NPs_ = 85.11% RSC _3,6 dihydroxyflavone, lutein, and selenium methyl selenocysteine_ = 70.63% > RSC _Au-3,6 dihydroxyflavone_ = 70.01% > RSC _lutein_ = 63.85% > RSC _3,6 dihydroxyflavone_ = 62.11% > RSC _selenium methyl selenocysteine_ = 41.62% H_2_O_2_:RSC _AA_ = 96.12% > RSC _Au-triplet NPs_ = 83.10% RSC _3,6 dihydroxyflavone, lutein, and selenium methyl selenocysteine_ = 71.35% > RSC _Au-3,6 dihydroxyflavone_ = 70.08% > RSC _lutein_ = 61.85% > RSC _3,6 dihydroxyflavone_ = 60.11% > RSC _selenium methyl selenocysteine_ = 40.02% ^●^NO: RSC _AA_ = 96.02% > RSC _Au-triplet NPs_ = 84.02% RSC _3,6 dihydroxyflavone, lutein, and selenium methyl selenocysteine_ = 69.09% > RSC _Au-3,6 dihydroxyflavone_ = 69.01% > RSC _3,6 dihydroxyflavone_ =61.24% > RSC_lutein_ = 60.85% > RSC _selenium methyl selenocysteine_ = 42.11%	[105]
8	Lignin Capped Silver NPs (LCSN)	DPPH (^●^N)	^●^N (DPPH method): RSC = 70%, IC_50_ = 3360 μg/mL	[170]
9	IONP@GA	DPPH (^●^N)	^●^N (DPPH method): RSC _IONP@GA3_ = 78% > RSC _IONP_ = 50%, IC_50 IONP@GA3_= 1.00 ± 0.003 mg/mL > IC_50 IONP_ = 4.7 ± 0.002 mg/mL	[23]
10	^a^ Ag-Se bimetallic	DPPH (^●^N), ABTS^●+^	^●^N (DPPH method): RSC _Trolox_ = 86.52 ± 0.12% > RSC _Ag–Se NPs_ = 59 ± 0.32%, IC_50 Trolox_ = 22.19 μg/mL < IC_50 Ag–Se_ NPs = 31 μg/mL ABTS^●+^: RSC _AA_ = 76.65 ± 0.29% > RSC _Ag–Se NPs_ = 62.54 ± 0.21%, IC_50 AA_ = 53.40μg/mL < IC_50Ag–Se_ NPs = 66.38 μg/mL	[33]
11	^a,f^ SiO_2_-coated Ag nanoparticles	DPPH (^●^N)	^●^N (DPPH method): Fast phase _SiO2-coated Ag_ n = 2 BDE _SiO2-coated Ag_ decreases by 2 kcal/mol	[166]
12	ZnO@CA NPs	ABTS^●+^	ABTS^●+^: RSC _CA_ = 93.25 ± 0.43% > RSC _ZnO@CA NPs_ = 73.68 ± 2.51%	[25]
13	poly(lactic-co-glycolic acid) (PLGA) NPs coated with polysorbate 80 (PS80) gallic acid	ABTS^●+^	ABTS^●+^: RSC _GA_ > RSC _NP-PLGA-GA_ > RSC _NP-PLGA/PS80-GA_	[167]
14	BSA-CA	DPPH (^●^N)	^●^N (DPPH method): RSC _CA_ = 91.9% > RSC _BSA-CA_ = 89.7% > RSC_BSA_ = 9.0%, RP: RP_CA_ >> RP _BSA-CA_ = 0.662 > RP _BSA_ = 0.010 ORAC: ORAC_CA_ = 4823.5 > ORAC _BSA-CA_ = 4073.9 > ORAC _BSA_ = 546.4	[22]
15	^d^ ACSSNs-CA	DPPH (^●^N)	^●^N (DPPH method): RSC _CA_ ≈ 95% > RSC_ACSSNs-CA_ ≈ 85%/Chelating Activity (CA): CA _ACSSNs-CA_ ≈ 97% > CA _CA_ ≈ 25%/^1^O_2_:k_q ACSSNs-CA_ = 1.3 × 10^6^ M^−1^·s^−1^> k_qCA_ = 4.6 × 10^5^ M^−1^·s^−1^	[19]
16	^a^ Se@Trolox	ABTS^●+^	ABTS^●+^: Se@Trolox > Trolox and Se@MUN	[37]
17	Au@PEG (Au@Trolox)	DPPH (^●^N)	^●^N (DPPH): Au@Trolox > Au@PEG + Trolox ≈ Trolox	[162]
18	ZnO@GA	ABTS^●+^	ABTS^●+^: RSC _GA_ ≈ 93.25 ± 0.43% > RSC_ZnO@GA_ = 69.71 ± 5.26%	[168]
19	ACSSNPs-CA (Carminic acid)	^1^O_2_	^1^O_2_: K_T ACSSNPs-CA_ = 1.30 × 10^8^ M^−1^ s ^−1^ > k_TCA_ = 6.35 × 10^7^ M^−1^·s^−1^ D_2_O: K_T ACSSNPs-CA_ 1.67 × 10^8^ M^−1^ s ^−1^ > k_T CA_ = 1.46 × 10^7^ M^−1^·s^−1^	[28]
20	^a^ SiO_2_-HALP NPs	DPPH (^●^N)	^●^N (DPPH): n_scavenged_ = SiO_2_[A300]-HALP >> SiO_2_[A90]-HALP >> HALP >> SiO_2_[OX50]-HALP > SiO_2_[S300]-HALP	[29]
21	^a^ GLA@SiO_2_@GLAM, SiO_2_@GLA, SiO_2_@GLAM	DPPH (^●^N)	^●^N (DPPH): Ea (kJ/mol (±1)) Ea_{GLA@SiO2@GLAM} [2:1]_= 42.2 > Ea_{GLA@SiO2@GLAM} [3:1]_= 46.6 > Ea_SiO2@GLA_= 65.7 > Ea_SiO2@GLAM_ = 123.3	[15]
22	^a^ HNT-Trolox/Que	DPPH (^●^N), ROO^●^	^●^N (DPPH): n_Que_ = 4.0 ±0.2 > n_HNT/Que_ = 3.8 ± 0.2> n_HNT-Trolox/Que_ = 2.8 ±0.2 > n_Trolox_ = 2.0 ± 0.2> n_HNT-Trolox_ = 1.3 ± 0.2	[163]
23	C-SNPs, EC-SNPs, EGCG-SNPs, PAG-SNPs	DPPH (^●^N)	^●^N (DPPH): IC_50EGCG-SNPs_ = 0.59± 0.02μg/mL > IC_50 EC-SNPs_ = 0.54 ± 0.05 μg/mL > IC_50 EGCG_ = 0.52 ± 0.04 μg/mL > IC_50 EC_ = 0.50 ± 0.04 μg/mL > IC_50 PAC-SNPs_ = 0.24 ± 0.04 μg/mL > IC_50 PAC_ = 0.23 ± 0.03 μg/mL > IC_50 C_ = 0.22 ± 0.02 μg/mL > IC_50 C-SNPs_ = 0.21 ± 0.03 μg/ml	[111]
24	PLA-UA NPs	HOCl	Antioxidants decrease the oxidation of TMB by HOCl	[100]
25	PLGA-Que NPs	DPPH (^●^N)	^●^N (DPPH): RSC_F3_ = 80%> RSC_F2_ = 79%	[34]
26	PCL-Que NPs	DPPH (^●^N), O_2_^●−^	^●^N (DPPH): EC_50Quercetin-biapigenin_ = 5.95 ± 0.97 μg/mL > EC_50 Quercetin-biapigenin PCL-loaded nanoparticles_ = 5.73 ± 1.20 μg/mL/O_2_^●−^: EC_50Quercetin-biapigenin_ = 72.71 ± 4.07μg/mL/Iron (II) chelating: EC_50Quercetin-biapigenin_ = 11.56 ± 0.44 μg/mL < EC_50 Quercetin-biapigenin PCL-loaded nanoparticles_ = 23.50 ± 0.55μg/ml	[164]
27	Vitamin E, catechol, and Ag NPs from *Hibiscus rosasinensis (HRS)* extracts within a chitosan matrix	DPPH(^●^N), H_2_O_2_, ^●^NO	^●^N (DPPH method): IC_50Cs–AA–Glu_ = 13.38 ± 4.7 μg/mL/^●^NO: IC_50Cs–AA–Glu_ = 1.19 ± 1.82%	[41]
28	^a,e^ AMSN-RA	DPPH(^●^N)	^●^N (DPPH method): max RSC_AMSN-RA_ ≈ 97% > RSC_RA_ ≈ 83%	[172]
29	SiO_2_-Que	O_2_^●−^	O_2_^●−^: RSC _quercetin_ = over 90% > RSC_SiO2-Que_ = 73%	[165]
30	^b,c^ V_2_O_5_@pDA@MnO_2_	ROS	Enzyme-mimicking antioxidant effect (GPx-like)	[174]
31	Nanohybrid HNT/AH_2_	DPPH(^●^N), ROO^●^	DPPH method: HNT/AH_2_ 290% vs. asc. acid (MeOH)/reaction with ROO^●:^ rate constant 5.1 × 104 M^−1^ s^−1^	[171]
32	Fe_3_O_4_@PDA-CuCl_2_	DPPH (^●^N)	^●^N (DPPH method): IC_50Fe3O4@PDA_ = 258μg/mL < IC_50 BHT_ = 386 μg/mL < IC_50 Fe3O4@PDA-CuCl2_ = 450 mg/mL	[175]
33	Iron oxide NPs (SPION) capped with GA, Trolox, and nordihydroguaiaretic acid	ROO^•^	O_2_ consumption ((−d[O_2_]/dt/µMs^−1^): MAG-GA,MAG-NDGA = 1.3 ± 0.2 > MAG-TX = 1.3 ± 0.2)	[58]

^a^ Hydrogen Atom Transfer (HAT) proposed mechanism, ^b^ SOD-mimetic activity, ^c^ CAT-mimetic activity, ^d^ SPLET, ^e^ SET, ^f^ PCET suggested by authors in each case. *Antioxidant efficiency is listed as and where referred to by authors.

## 6. Conclusions

Nanoantioxidants comprise an essential part of utilized materials, especially in the bio-medical, pharmaceutical, and cosmeceutical industries. Inspiration from natural antioxidant systems, in combination with the unique properties of the nanoscale, has paved the way for the development of optimized artificial nanoantioxidant systems. Nanotechnology and nanoengineering are mainly utilized to improve stability, control release, and overcome toxicity and biocompatibility issues, and thus facilitate industrial production.

Interestingly, specific key aspects indicate that nanoengineering (i) can induce antioxidant properties to an initially inactive component, (ii) can promote synergistic effects between the {organic–inorganic components} of the hybrid system, and (iii) can control the properties of support materials (e.g., minimizing its toxicity). The current research and industrial interest in nanoantioxidants are additionally reflected in the number of available experimental evaluation methods and the extensive use of specific techniques described in this research.

Nanoantioxidants can be prepared through different engineering concepts, such as direct extraction from natural sources, wet chemistry, or flame synthesis. Post-synthetical treatments such as covalent and non-covalent surface functionalization of nanoparticles are widely used nanoengineering concepts in tuning physicochemical characteristics. Nanoantioxidants, however, share the need for strict control of their toxicity, often regulated by the synthetic pathway, since nanostructures are more potent for inducing adverse effects on human health and the ecosystem.

Despite being recently-introduced, there is adequate evidence that artificial nanoantioxidants operate through mechanisms utilized in natural systems, such as hydrogen atom transfer (HAT) or electron transfer mechanisms. We expect that the vital information covered in this work will constructively organize the overall knowledge regarding antioxidants, and hopefully clarify specific knowledge gaps, facilitating their bio-medical/biotechnological application potential.

## Figures and Tables

**Table 1:** List of common free radicals.

Oxygen-Centered Radicals		Non-Oxygen-Centered Radicals	
*Hydroxyl radical*	*^•^*OH	*Nitric oxide radical*	^•^NO
*Superoxide anion radical*	^•^O_2_^−^	*Nitrogen dioxide*	^•^NO_2_
*Peroxyl radicals*	HO_2_^•^, ROO^•^, LOO^•^	*Carbon monoxide anion*	CO^•−^
*Alkoxyl/Phenoxyl radicals*	RO^•^, LO^•^, Tyr^•^	*Trisulfur radical*	S_3_^•−^
*Semiquinone radical*	SQ^•−^	*Chlorine radicals*	Cl^•^, Cl_2_^•−^
*Carbonate radical*	CO_3_^•^		
*Sulfate/Phosphate radical*	SO_4_^•−^, PO_4_^2−•^		

**Table 2:** List of engineered nanoantioxidants synthesized directly from natural sources.

	Nanoantioxidant	Target	Evaluation Methods/Antioxidant Efficiency *	Ref.
1	Ag NPs from *clerodendrum phlomidis* leaf extract	^●^N, O_2_^●−^	^●^N (DPPH method): IC_50_ = 55.86 μg/mL < IC_50_ = 202.2 μg/mL O_2_^●−^: IC_50_ = 9.12 μg/mL < IC_50_ = 182.8 μg/mL FRAP: 1.63 AU < FA ≈ 1.8 AU Phosphomolybdate assay: 910 AEAA > FA ≈ 710 AEAA	[119]
2	SLG/GA	DPPH (^●^N), ^●^OH	enhanced the antioxidant activity of phenolic acids	[40]
3	Ag NPs, *Malus domestica*	DPPH (^●^N)	DPPH method/RSC = 75.16%	[122]
4	Ag NPs, *Asphodelus aestivus Brot.*	DPPH (^●^N), ABTS^●+^, H_2_O_2_	^●^N (DPPH): RSC _Ag NPs_ = 67.54 ± 5.49 > RSC _ASP_ = 31.82 ± 4.04 ABTS^●+^: RSC _Ag NPs_ = 79.94 ± 0.02 > RSC _ASP_ = 39.62 ± 0.02 H_2_O_2_/RSC _Ag NPs_ = 31.67 ± 0.06 < RSC _ASP_ = 55.86 ± 0.14	[123]
5	^a^ Ag NPs, *Lippia Nodiflora* (*ASP*)	DPPH (^●^N), O_2_^●−^, ^●^OH	^●^N: (DPPH method)/RSC _Ag NPs_ = 67% < RSC _BHT_ = 83% O_2_^●−^: RSC _Ag NPs_ = 70% < RSC _BHT_ = 84% ^●^OH: RSC _Ag NPs_ = 69% < RSC _BHT_ = 75% Reducing power: RSC _Ag NPs_ = 0.115 < RSC _BHT_ = 0.095 H_2_O_2_: RSC _Ag NPs_ = 71.1% > RSC _BHT_ = 68.2%	[124]
6	Ag NPs, *Memecylon umbellatum Burm*	DPPH (^●^N), O_2_^●−^	^●^N: (DPPH method): RSC _Ag NPs_ = 81.57% < RSC _BHT_ = 85.39%, EC_50 Ag NPs_ = 53.46 μg/mL > EC_50 BHT_ = 37.92 μg/mL O_2_^●−^: RSC _Ag NPs_ = 74.76% < RSC _BHT_ = 80.71%, EC_50 Ag NPs_ = 66.68 μg/mL > EC_50 BHT_ = 53.39 μg/mL	[125]
7	^a^*Ct Ag NPs*, *Calophyllum tomentosum*	DPPH (^●^N), H_2_O_2,_ ^●^NO	^●^N: (DPPH method): RSC _CtAg NPs_ = 90% > RSC_BHT_ H_2_O_2_: RSC _CtAg NPs_ = 83.94% > RSC _AA_ ON^●^: RSC _CtAg NPs_ = 78.46% < RSC_BHT_ = 79.11% Reducing power: RSC _CtAg NPs_ = 74% < RSC_BHT_ = 83%	[126]
8	^a^ Ag NPs, *Morus alba* (*Mulberry*)	DPPH, ABTS^+●^, O_2_^●−^, ^●^NO, Metal chelation	^●^N: (DPPH method): IC_50 AgNPs_ = 97.273 μg/mL < IC_50 plant extract_ = 143.967 μg/mL ABTS^+^: IC_50 AgNPs_ = 25.929 μg/mL < IC_50 plant extract_ = 53.832 μg/mL O_2_^●−^: IC_50 AgNPs_ = 37.097 μg/mL < IC_50 plant extract_ = 77.479 μg/mL ON^●^: IC_50 AgNPs_ = 70.992 μg/mL < IC_50 plant extract_ = 101.587 μg/mL Metal chelation: IC_50 AgNPs_ = 54.325 μg/mL < IC_50 plant extract_ = 73.837 μg/mL	[127]
9	^a^ AuNPs, from KG, *Lotus leguminosae*	DPPH (^●^N)	^●^N: (DPPH method): EC_50 GA_ = 11.92 μg/mL > EC_50 Au NPs_ = 30.54 μg/mL > EC_50 KG_ = 48.9 μg/ml	[128]
10	Au, Ag NPs, *Plumbago zeylanica*	DPPH (^●^N)	^●^N: (DPPH method): RSC_AuNPs_ = 87.34% > RSC_AgNPs_ = 78.17% > RSC_BHT_ = 74.88% > RSC_extract_ = 71.16%	[129]
11	Ti-Pt NPs from *Tragia involucrata*	DPPH (^●^N)	^●^N (DPPH method): RSC_Ti-Pt NPs_ = 64 ± 0.43% > RSC_AE-Ti_ Reducing Power (RP): RSC_Ti-Pt NPs_ = 13.45 ± 0.23% > RSC_AE-Ti_ Total Antioxidant Properties:RSC_Ti-Pt NPs_ = 15.85 ± 0.22% > RSC_AE-Ti_	[130]
12	^a^ Cu NPs*, Falcaria vulgaris*	DPPH (^●^N)	^●^N (DPPH method): IC_50*F.Vulgaris*_ = 392 μg/mL > IC_50 BHT_ = 314 μg/mL > IC_50 CuNPs_ = 190 μg/ml	[131]
13	Cu NPs, *Borreria hispida* (*Linn.*)	DPPH (^●^N)	^●^N (DPPH method): IC_50 crude extract_ = 1.5 μg/mL > IC_50 CuNPs_ = 0.6 μg/mL.	[132]
14	Ag/Cu, Cu/Zn NPs, *Borassus flabellife*	DPPH (^●^N), ^●^OH, H_2_O_2_	^●^N (DPPH method): C = 100 μg/mL RSC_AA_ = 72% > RSC_Ag/CuNPs_ = 58% > RSC_Cu/ZnNPs_ = 40% ^●^OH: C = 100 μg/mL, RSC_AA_ = 74% > RSC_Ag/CuNPs_ = 48% > RSC_Cu/ZnNPs_ = 38% H_2_O_2_: C = 100 μg/mL, RSC_AA_ = 74% > RSC_Ag/CuNPs_ = 42% > RSC_Cu/ZnNPs_ = 28%	[133]
15	AgPt NPs, *Vernonia mespilifolia plant*	DPPH (^●^N), ABTS^+●^	^●^N (DPPH method): IC_50 AA_ = 131.8 ± 0.4 μg/mL > IC_50 AgNPs_ = 28.5 ± 0.1 μg/mL > IC_50 AgPt NPs_ = 19.5 ± 0.2 μg/mL ABTS^+●^: IC_50 AgNPs_ = 302.7 ± 2.8 μg/mL > IC_50 AA_ = 210.7 ± 1.0 μg/mL > IC_50 AgPt NPs_ = 21.6 ± 2.1 μg/mL FRAP_AgPt NPs_ = 44.1 ± 2.7 mg GAE/g > FRAP _AgNPs_ = 18.5 ± 0.2 mg GAE/g	[134]
16	Au/Ag (BM NPs), *Clove buds*	DPPH (^●^N), ABTS^+●^, ^●^OH	^●^N (DPPH method): IC_50 Au/Ag BMNPs_= 0.5 IC_50 AgNPs_ ABTS^+●^: IC_50_ = 18.27 μg/mL. ^●^OH:IC_50_ = 30.59 μg/ml	[135]
17	ZnO NPs, *Cucurbita seed*	DPPH (^●^N)	^●^N (DPPH method): RSC_ZnONPs_ = 91.37 ± 6.39% > RSC_AA_ = 83.68 ± 5.85%, IC_50AA_ = 45.33 μg/mL > IC_50ZnONPs_ = 40.81 μg/ml	[136]
18	MONPs (Magnesium oxide), *Pisonia Alba*	DPPH (^●^N)	^●^N (DPPH method): RSC = 65%/FRAP: RSC = 69.3%	[137]
19	ZnO NPs, *Tecoma castanifolia leaf*	DPPH (^●^N)	^●^N (DPPH method): RSC = 67%, at 100 μg/mL	[138]
20	ZnO NPs, *Knoxia sumatrensis* aqueous (Ks-ALE)	DPPH (^●^N), ABTS^+●^, H_2_O_2_	^●^N (DPPH method): IC_50_ = 95.80 μg/mL ABTS^+●^: IC_50_ = 92.29 μg/mL/H_2_O_2_: IC_50_ = 98.92 μg/ml	[139]
21	CuNPs, *Cissus vitiginea*	DPPH (^●^N)	^●^N (DPPH method): C = 80 μg/mL, RSC_AA_ = 90.31 ± 6.32% >RSC_CuONPs_ = 86.78 ± 6.07% > RSC*_Cissus Vitiginea_* = 82.37 ± 5.76%, IC_50 *Cissus Vitiginea*_ = 50.51μg/mL > IC_50 CuONPs_ = 45.29 μg/mL > IC_50 AA_ = 41.33 μg/ml	[140]
22	TiO_2_ NPs, *Cola nitida*	DPPH (^●^N)	^●^N (DPPH method): RSC = 60.08%/H_2_O_2_: RSC = 99.23%	[141]
23	^b^ CeO_2_ NPs, *Stachys japonica*	DPPH (^●^N), ABTS^+●^	^●^N (DPPH method): IC_50_ = 109.5 ± 0.26 μg/mL ABTS^+●^: IC_50_ = 12.16 ± 0.12 μg/ml	[142]
24	^b^ CeONP, *Aloe Vera*	DPPH (^●^N)	^●^N (DPPH method): RSC ≈ 83%	[143]

^a^ Hydrogen atom transfer (HAT) proposed mechanism, ^b^ SOD-mimetic activity suggested. ∗ Antioxidant efficiency is listed as and where referred to by authors.

## Data Availability

Not applicable.

## Abbreviations

**ROS**—Reactive Oxygen Species, **RNS**—Reactive Nitrogen Species, **NPs**—Nanoparticles, **NMs—**Nanomaterials, **HAT**—Hydrogen Atom Transfer, **PCET**—Proton-Coupled Electron Transfer, **SET-PT**—Single Electron Transfer-Proton Transfer, **SPLET**—Sequential Proton loss electron transfer, **RSC—**Radical Scavenging Capacity, **EPR**—Electron Paramagnetic Resonance, **DPPH**—2,2-diphenyl-1-picrylhydrazyl, **ABTS**—2,2′-Azinobis-(3-Ethylbenzthia-zolin-6-Sulfonic Acid, **FRAP**—Ferric-reducing antioxidant power, **ORAC—**Oxygen Radical Absorbance Capacity, **TRAP**—Trapping Antioxidant Parameter, **TEAC—**Trolox Equivalent Antioxidant Capacity, **FC**—Total Phenol Content Folin-Ciocalteu, **TAC—**Total Antioxidant Activity, TRP—Total Reducing Power, EC50**_/_**IC50-half-maximal Effective Concentration/Inhibitory Concentration, **SQ**—Semiquinone, **PDS**—Peroxydisulfate, **GPx**—Glutathione peroxidase, **CAT**—Catalase, **SOD**—Superoxide dismutase, **GA**—Gallic acid, **RA**—Rosmarinic acid, **Hya**—Hyaluronic acid, **CA**—Caffeic acid, **HALP**—Humic Acid-like Polycondensate, **PVA**—Poly(vinyl alcohol).

## References

[1] Shah S.T., Chowdhury Z.Z., Simarani K., Basirun W.J., Badruddin I.A., Hussien M., Alrobei H., Kamangar S. (2022). Nanoantioxidants: The Fourth Generation of Antioxidants—Recent Research Roadmap and Future Perspectives. Coatings.

[2] Halliwell B. (1996). Antioxidants in Human Health and Disease. Annu. Rev. Nutr..

[3] Zeb A. (2020). Concept, mechanism, and applications of phenolic antioxidants in foods. J. Food Biochem..

[4] Pham-Huy L.A., He H., Pham-Huy C. (2008). Free radicals, antioxidants in disease and health. Int. J. Biomed. Sci..

[5] Liu R., Mabury S.A. (2020). Synthetic Phenolic Antioxidants: A Review of Environmental Occurrence, Fate, Human Exposure, and Toxicity. Environ. Sci. Technol..

[6] Farah F., Farah F.H. (2019). Nanocarriers As Delivery Systems for Therapeutics Agents. Int. J. Pharm. Sci. Res..

[7] Gil D., Rodriguez J., Ward B., Vertegel A., Ivanov V., Reukov V. (2017). Antioxidant activity of SOD and catalase conjugated with nanocrystalline ceria. Bioengineering.

[8] Flieger J., Flieger W., Baj J. (2021). Antioxidants: Classification, Natural Sources, Activity / Capacity. Materials.

[9] Ahmad F., Salem-Bekhit M.M., Khan F., Alshehri S., Khan A., Ghoneim M.M., Wu H.F., Taha E.I., Elbagory I. (2022). Unique Properties of Surface-Functionalized Nanoparticles for Bio-Application: Functionalization Mechanisms and Importance in Application. Nanomaterials.

[10] Baig N., Kammakakam I., Falath W., Kammakakam I. (2021). Nanomaterials: A review of synthesis methods, properties, recent progress, and challenges. Mater. Adv..

[11] Hasanuzzaman M., Bhuyan M.H.M.B., Zulfiqar F., Raza A., Mohsin S.M., Al Mahmud J., Fujita M., Fotopoulos V. (2020). Reactive oxygen species and antioxidant defense in plants under abiotic stress: Revisiting the crucial role of a universal defense regulator. Antioxidants.

[12] Khalil I., Yehye W.A., Etxeberria A.E., Alhadi A.A., Dezfooli S.M., Julkapli N.B.M., Basirun W.J., Seyfoddin A. (2020). Nanoantioxidants: Recent trends in antioxidant delivery applications. Antioxidants.

[13] Kumar H., Bhardwaj K., Nepovimova E., Kuča K., Dhanjal D.S., Bhardwaj S., Bhatia S.K., Verma R., Kumar D. (2020). Antioxidant functionalized nanoparticles: A combat against oxidative stress. Nanomaterials.

[14] Diniz L.R.L., Da Silva Maia Bezerra Filho C., Fielding B.C., De Sousa D.P., Gil G. (2020). Natural Antioxidants: A Review of Studies on Human and Animal Coronavirus. Oxid. Med. Cell. Longev..

[15] Theofanous A., Sarli I., Fragou F., Bletsa E., Deligiannakis Y., Louloudi M. (2022). Antioxidant Hydrogen-Atom-Transfer to DPPH Radicals by Hybrids of {Hyaluronic-Acid Components}@SiO_2_. Langmuir.

[16] Mayer J.M., Hrovat D.A., Thomas J.L., Borden W.T. (2002). Proton-coupled electron transfer versus hydrogen atom transfer in benzyl/toluene, methoxyl/methanol, and phenoxyl/phenol self-exchange reactions. J. Am. Chem. Soc..

[17] Anwar H., Hussain G., Mustafa I. (2018). Antioxidants from Natural Sources. Antioxidants Foods Its Appl..

[18] Vaiserman A., Koliada A., Zayachkivska A., Lushchak O. (2020). Nanodelivery of Natural Antioxidants: An Anti-aging Perspective. Front. Bioeng. Biotechnol..

[19] Arriagada F., Günther G., Nos J., Nonell S., Olea-Azar C., Morales J. (2019). Antioxidant nanomaterial based on core–shell silica nanospheres with surface-bound caffeic acid: A promising vehicle for oxidation-sensitive drugs. Nanomaterials.

[20] Khojasteh A., Mirjalili M.H., Alcalde M.A., Cusido R.M., Eibl R., Palazon J. (2020). Powerful plant antioxidants: A new biosustainable approach to the production of rosmarinic acid. Antioxidants.

[21] Platzer M., Kiese S., Herfellner T., Schweiggert-Weisz U., Miesbauer O., Eisner P. (2021). Common trends and differences in antioxidant activity analysis of phenolic substances using single electron transfer based assays. Molecules.

[22] Fan Y., Liu Y., Gao L., Zhang Y., Yi J. (2018). Improved chemical stability and cellular antioxidant activity of resveratrol in zein nanoparticle with bovine serum albumin-caffeic acid conjugate. Food Chem..

[23] Shah S.T., Yehye W.A., Saad O., Simarani K., Chowdhury Z.Z., Alhadi A.A., Al-Ani L.A. (2017). Surface functionalization of iron oxide nanoparticles with gallic acid as potential antioxidant and antimicrobial agents. Nanomaterials.

[24] Badhani B., Sharma N., Kakkar R. (2015). RSC Advances. R. Soc. Chem. Adv..

[25] Choi K.H., Nam K.C., Lee S.Y., Cho G., Jung J.S., Kim H.J., Park B.J. (2017). Antioxidant potential and antibacterial efficiency of caffeic acid-functionalized ZnO nanoparticles. Nanomaterials.

[26] Cao H., Cheng W.X., Li C., Pan X.L., Xie X.G., Li T.H. (2005). DFT study on the antioxidant activity of rosmarinic acid. J. Mol. Struct. Theochem.

[27] Sahiner N., Sagbas S., Aktas N. (2016). Preparation and characterization of monodisperse, mesoporous natural poly(tannic acid)-silica nanoparticle composites with antioxidant properties. Microporous Mesoporous Mater..

[28] Arriagada F., Ugarte C. (2020). Carminic Acid Linked to Silica Nanoparticles as Pigment / Antioxidant Bifunctional Excipient for Pharmaceutical Emulsions. Pharmaceutics.

[29] Bletsa E., Stathi P., Dimos K., Louloudi M., Deligiannakis Y. (2015). Interfacial Hydrogen Atom Transfer by nanohybrids based on Humic Acid Like Polycondensates. J. Colloid Interface Sci..

[30] Shah B.R., Zhang C., Li Y., Li B. (2016). Bioaccessibility and antioxidant activity of curcumin after encapsulated by nano and Pickering emulsion based on chitosan-tripolyphosphate nanoparticles. Food Res. Int..

[31] Menaga D., Rahman P.K.S.M., Rajakumar S., Ayyasamy P.M. (2021). Antioxidant and Cytotoxic Activities of A Novel Isomeric Molecule (PF5) Obtained from Methanolic Extract of Pleurotus Florida Mushroom. J. Bioresour. Bioprod..

[32] Arriagada F., Correa O., Günther G., Nonell S., Mura F., Olea-Azar C., Morales J. (2016). Morin flavonoid adsorbed on mesoporous silica, a novel antioxidant nanomaterial. PLoS ONE.

[33] Mittal A.K., Kumar S., Banerjee U.C. (2014). Quercetin and gallic acid mediated synthesis of bimetallic (silver and selenium) nanoparticles and their antitumor and antimicrobial potential. J. Colloid Interface Sci..

[34] Anwer M.K., Al-Mansoor M.A., Jamil S., Al-Shdefat R., Ansari M.N., Shakeel F. (2016). Development and evaluation of PLGA polymer based nanoparticles of quercetin. Int. J. Biol. Macromol..

[35] Ebabe Elle R., Rahmani S., Lauret C., Morena M., Bidel L.P.R., Boulahtouf A., Balaguer P., Cristol J.P., Durand J.O., Charnay C. (2016). Functionalized Mesoporous Silica Nanoparticle with Antioxidants as a New Carrier That Generates Lower Oxidative Stress Impact on Cells. Mol. Pharm..

[36] Das S., Batuta S., Alam M.N., Fouzder C., Kundu R., Mandal D., Begum N.A. (2017). Antioxidant flavone analog functionalized fluorescent silica nanoparticles: Synthesis and exploration of their possible use as biomolecule sensor. Colloids Surfaces B Biointerfaces.

[37] Li Y., Li X., Zheng W., Fan C., Zhang Y., Chen T. (2013). Functionalized selenium nanoparticles with nephroprotective activity, the important roles of ROS-mediated signaling pathways. J. Mater. Chem. B.

[38] Nie Z., Liu K.J., Zhong C.J., Wang L.F., Yang Y., Tian Q., Liu Y. (2007). Enhanced radical scavenging activity by antioxidant-functionalized gold nanoparticles: A novel inspiration for development of new artificial antioxidants. Free Radic. Biol. Med..

[39] Brown M.B., Jones S.A. (2005). Hyaluronic acid: A unique topical vehicle for the localized delivery of drugs to the skin. J. Eur. Acad. Dermatology Venereol..

[40] Li X., Li M., Liu J., Ji N., Liang C., Sun Q., Xiong L. (2017). Preparation of Hollow Biopolymer Nanospheres Employing Starch Nanoparticle Templates for Enhancement of Phenolic Acid Antioxidant Activities. J. Agric. Food Chem..

[41] Nayak D., Minz A.P., Ashe S., Rauta P.R., Kumari M., Chopra P., Nayak B. (2016). Synergistic combination of antioxidants, silver nanoparticles and chitosan in a nanoparticle based formulation: Characterization and cytotoxic effect on MCF-7 breast cancer cell lines. J. Colloid Interface Sci..

[42] Huq T., Khan A., Brown D., Dhayagude N., He Z., Ni Y. (2022). Sources, production and commercial applications of fungal chitosan: A review. J. Bioresour. Bioprod..

[43] Bharathi D., Ranjithkumar R., Vasantharaj S., Chandarshekar B., Bhuvaneshwari V. (2019). Synthesis and characterization of chitosan/iron oxide nanocomposite for biomedical applications. Int. J. Biol. Macromol..

[44] Qiu Y., Wang Z., Owens A.C.E., Kulaots I., Chen Y., Kane A.B., Hurt R.H. (2014). Antioxidant chemistry of graphene-based materials and its role in oxidation protection technology. Nanoscale.

[45] Rodríguez-Varillas S., Fontanil T., Obaya Á.J., Fernández-González A., Murru C., Badía-Laíño R. (2022). Biocompatibility and Antioxidant Capabilities of Carbon Dots Obtained from Tomato (*Solanum lycopersicum*). Appl. Sci..

[46] Liberti D., Alfieri M.L., Monti D.M., Panzella L., Napolitano A. (2020). A melanin-related phenolic polymer with potent photoprotective and antioxidant activities for dermo-cosmetic applications. Antioxidants.

[47] De Goncalves R.C.R., Pombeiro-Sponchiado S.R. (2005). Antioxidant activity of the melanin pigment extracted from Aspergillus nidulans. Biol. Pharm. Bull..

[48] P’yanova L.G., Drozdov V.A., Sedanova A.V., Drozdetskaya M.S., Glyzdova M.V., Kravchenko E.A. (2018). Synthesis of Modified Carbon Sorbents and a Study of Their Antioxidant Properties. Prot. Met. Phys. Chem. Surfaces.

[49] Skrypnik L., Babich O., Sukhikh S., Shishko O., Ivanova S., Mozhei O., Kochish I., Nikonov I. (2021). A study of the antioxidant, cytotoxic activity and adsorption properties of karelian shungite by physicochemical methods. Antioxidants.

[50] Sajo M.E.J., Kim C.S., Kim S.K., Shim K.Y., Kang T.Y., Lee K.J. (2017). Antioxidant and Anti-Inflammatory Effects of Shungite against Ultraviolet B Irradiation-Induced Skin Damage in Hairless Mice. Oxid. Med. Cell. Longev..

[51] Seal S., Jeyaranjan A., Neal C.J., Kumar U., Sakthivel T.S., Sayle D.C. (2020). Engineered defects in cerium oxides: Tuning chemical reactivity for biomedical, environmental, & energy applications. Nanoscale.

[52] Nelson B.C., Johnson M.E., Walker M.L., Riley K.R., Sims C.M. (2016). Antioxidant cerium oxide nanoparticles in biology and medicine. Antioxidants.

[53] Sharpe E., Andreescu D., Andreescu S. (2011). Artificial nanoparticle antioxidants. ACS Symp. Ser..

[54] Matter M.T., Furer L.A., Starsich F.H.L., Fortunato G., Pratsinis S.E., Herrmann I.K. (2019). Engineering the Bioactivity of Flame-Made Ceria and Ceria/Bioglass Hybrid Nanoparticles. ACS Appl. Mater. Interfaces.

[55] Caputo F., Mameli M., Sienkiewicz A., Licoccia S., Stellacci F., Ghibelli L., Traversa E. (2017). A novel synthetic approach of cerium oxide nanoparticles with improved biomedical activity. Sci. Rep..

[56] Datta A., Mishra S., Manna K., Saha K.D., Mukherjee S., Roy S. (2020). Pro-Oxidant Therapeutic Activities of Cerium Oxide Nanoparticles in Colorectal Carcinoma Cells. ACS Omega.

[57] He X., Aker W.G., Fu P.P., Hwang H.M. (2015). Toxicity of engineered metal oxide nanomaterials mediated by nano-bio-eco-interactions: A review and perspective. Environ. Sci. Nano.

[58] Scurti S., Caretti D., Mollica F., Di Antonio E., Amorati R. (2022). Chain-Breaking Antioxidant and Peroxyl Radical Trapping Activity of Phenol-Coated Magnetic Iron Oxide Nanoparticles. Antioxidants.

[59] Viglianisi C., Scarlini A., Tofani L., Menichetti S., Baschieri A., Amorati R. (2019). Magnetic nanoantioxidants with improved radical-trapping stoichiometry as stabilizers for inhibition of peroxide formation in ethereal solvents. Sci. Rep..

[60] Feng L., Liu Z. (2016). Biomedical Applications of Carbon Nanomaterials. Biomed. Appl. Toxicol. Carbon Nanomater..

[61] Fragou F., Stathi P., Deligiannakis Y., Louloudi M. (2022). Safe-by-Design Flame Spray Pyrolysis Nano-SiO_2_: Minimizing the ROS generation and acute toxicity by design. ACS Appl. Nano Mater..

[62] Helberg J., Pratt D.A. (2021). Autoxidation vs. antioxidants-the fight for forever. Chem. Soc. Rev..

[63] Eftekhari A., Dizaj S.M., Chodari L., Sunar S., Hasanzadeh A., Ahmadian E., Hasanzadeh M. (2018). The promising future of nano-antioxidant therapy against environmental pollutants induced-toxicities. Biomed. Pharmacother..

[64] Phaniendra A., Jestadi D.B., Periyasamy L. (2015). Free Radicals: Properties, Sources, Targets, and Their Implication in Various Diseases. Indian J. Clin. Biochem..

[65] Çalişkan B., Çalişkan A.C. (2018). EPR Analysis of Antioxidant Compounds. Free Radicals, Antioxidants Dis..

[66] Nauser T., Gebicki J.M. (2019). Fast reaction of carbon free radicals with flavonoids and other aromatic compounds. Arch. Biochem. Biophys..

[67] Das B., Makol A., Kundu S. (2022). Phosphorus radicals and radical ions. Dalt. Trans..

[68] Ulas G., Lemmin T., Wu Y., Gassner G.T., DeGrado W.F. (2016). Designed metalloprotein stabilizes a semiquinone radical. Nat. Chem..

[69] Shafirovich V., Dourandin A., Huang W., Geacintov N.E. (2001). The Carbonate Radical Is a Site-selective Oxidizing Agent of Guanine in Double-stranded Oligonucleotides. J. Biol. Chem..

[70] Bühl M., Dabell P., Manley D.W., McCaughan R.P., Walton J.C. (2015). Bicarbonate and Alkyl Carbonate Radicals: Structural Integrity and Reactions with Lipid Components. J. Am. Chem. Soc..

[71] Stathi P., Fotou E., Moussis V., Tsikaris V., Louloudi M., Deligiannakis Y. (2022). Control of Tyrosyl Radical Stabilization by {SiO_2_@Oligopeptide} Hybrid Biomimetic Materials. Langmuir.

[72] Dixon W.T., Murphy D. (1976). Determination of the acidity constants of some phenol radical cations by means of electron spin resonance. J. Chem. Soc. Faraday Trans. 2 Mol. Chem. Phys..

[73] Karoui H., Hogg N., Fréjaville C., Tordo P., Kalyanaraman B. (1996). Characterization of sulfur-centered radical intermediates formed during the oxidation of thiols and sulfite by peroxynitrite: ESR-spin trapping and oxygen uptake studies. J. Biol. Chem..

[74] Xia X., Zhu F., Li J., Yang H., Wei L., Li Q., Jiang J., Zhang G., Zhao Q. (2020). A Review Study on Sulfate-Radical-Based Advanced Oxidation Processes for Domestic/Industrial Wastewater Treatment: Degradation, Efficiency, and Mechanism. Front. Chem..

[75] Martínez M.C., Andriantsitohaina R. (2009). Reactive nitrogen species: Molecular mechanisms and potential significance in health and disease. Antioxidants Redox Signal..

[76] Taub T., Ruthstein S., Cohen H. (2018). The involvement of carbon-centered radicals in the aging process of coals under atmospheric conditions: An EPR study. Phys. Chem. Chem. Phys..

[77] Lednor P.W., Versloot P.C. (1983). Radical-anion chemistry of carbon monoxide. J. Chem. Soc. Chem. Commun..

[78] Pokrovski G.S., Kokh M.A., Desmaele E., Laskar C., Bazarkina E.F., Borisova A.Y., Testemale D., Hazemann J.L., Vuilleumier R., Ferlat G. (2021). The trisulfur radical ion S_3_^•−^ controls platinum transport by hydrothermal fluids. Proc. Natl. Acad. Sci. USA.

[79] Lei Y., Lei X., Westerhoff P., Zhang X., Yang X. (2021). Reactivity of Chlorine Radicals (Cl•and Cl2•-) with Dissolved Organic Matter and the Formation of Chlorinated Byproducts. Environ. Sci. Technol..

[80] Wang Y., Branicky R., Noë A., Hekimi S. (2018). Superoxide dismutases: Dual roles in controlling ROS damage and regulating ROS signaling. J. Cell Biol..

[81] Nosaka Y., Nosaka A.Y. (2017). Generation and Detection of Reactive Oxygen Species in Photocatalysis. Chem. Rev..

[82] Rubio L., Pyrgiotakis G., Beltran-Huarac J., Zhang Y., Gaurav J., Deloid G., Spyrogianni A., Sarosiek K.A., Bello D., Demokritou P. (2019). Safer-by-design flame-sprayed silicon dioxide nanoparticles: The role of silanol content on ROS generation, surface activity and cytotoxicity. Part. Fibre Toxicol..

[83] Lushchak V.I. (2016). Free radicals, reactive oxygen species, oxidative stresses and their classifications Chemico-Biological Interactions Free radicals, reactive oxygen species, oxidative stress and its classification. Chem. Biol. Interact..

[84] Dad S., Bisby R.H., Clark I.P., Parker A.W. (2006). Europe PMC Funders Group Formation of singlet oxygen from solutions of vitamin E. Free Radic Res..

[85] Shahidi F., Zhong Y. (2015). Measurement of antioxidant activity. J. Funct. Foods.

[86] Cao C., Chen Y., Wu Y., Deumens E., Cheng H.P. (2011). OPAL: A Multiscale Multicenter Simulation Package Based on MPI-2 Protocol. Int. J. Quantum Chem..

[87] Capaldo L., Ravelli D. (2017). Hydrogen Atom Transfer (HAT): A Versatile Strategy for Substrate Activation in Photocatalyzed Organic Synthesis. European J. Org. Chem..

[88] Deligiannakis Y., Sotiriou G.A., Pratsinis S.E. (2013). Antioxidant and antiradical SiO_2_ nanoparticles covalently functionalized with gallic acid. Tech. Proc. 2013 NSTI Nanotechnol. Conf. Expo, NSTI-Nanotech 2013.

[89] Di Meo F., Lemaur V., Cornil J., Lazzaroni R., Duroux J.L., Olivier Y., Trouillas P. (2013). Free radical scavenging by natural polyphenols: Atom versus electron transfer. J. Phys. Chem. A.

[90] Hammes-Schiffer S. (2002). Comparison of hydride, hydrogen atom, and proton-coupled electron transfer reactions. ChemPhysChem.

[91] Katarina N.M. (2007). Mechanistic studies of phenolic antioxidants in reaction with nitrogen- and oxygen-centered radicals. J. Mol. Struct. Theochem..

[92] Mayer J.M. (2011). Understanding hydrogen atom transfer: From bond strengths to marcus theory. Acc. Chem. Res..

[93] Najafi M. (2014). On the antioxidant activity of the Ortho and Meta substituted Daidzein derivatives in the gas phase and solvent environment. J. Mex. Chem. Soc..

[94] Zobo Mfomo J., Bikele Mama D., Lissouck D., Younang E., N’sikabaka S., Mbouombouo Ndassa I., Mbaze Meva’à L. (2017). Thermodynamics-antioxidant activity relationships of some 4-benzylidenamino-4, 5-dihydro-1h-1,2,4-triazol-5-one derivatives: Theoretical evaluation. Int. J. Food Prop..

[95] Clément J.L., Ferré N., Siri D., Karoui H., Rockenbauer A., Tordo P. (2005). Assignment of the EPR spectrum of 5,5-dimethyl-1-pyrroline N-oxide (DMPO) superoxide spin adduct. J. Org. Chem..

[96] Diamantis D.A., Oblukova M., Chatziathanasiadou M.V., Gemenetzi A., Papaemmanouil C., Gerogianni P.S., Syed N., Crook T., Galaris D., Deligiannakis Y. (2020). Bioinspired tailoring of fluorogenic thiol responsive antioxidant precursors to protect cells against H_2_O_2_-induced DNA damage. Free Radic. Biol. Med..

[97] Boligon A.A. (2014). Technical Evaluation of Antioxidant Activity. Med. Chem..

[98] Alam M.N., Bristi N.J., Rafiquzzaman M. (2013). Review on in vivo and in vitro methods evaluation of antioxidant activity. Saudi Pharm. J..

[99] Youssef M.M. (2014). Methods for Determining the Antioxidant Activity: A Review. Alexandria J. Food Sci. Technol..

[100] Antônio E., Antunes O. (2017). dos, R.; de Araújo, I.S.; Khalil, N.M.; Mainardes, R.M. Poly(lactic acid) nanoparticles loaded with ursolic acid: Characterization and in vitro evaluation of radical scavenging activity and cytotoxicity. Mater. Sci. Eng. C.

[101] Fa M., Yang D., Gao L., Zhao R., Luo Y., Yao X. (2018). The effect of AuNP modification on the antioxidant activity of CeO_2_ nanomaterials with different morphologies. Appl. Surf. Sci..

[102] Silveri F., Della Pelle F., Scroccarello A., Mazzotta E., Di Giulio T., Malitesta C., Compagnone D. (2022). Carbon Black Functionalized with Naturally Occurring Compounds in Water Phase for Electrochemical Sensing of Antioxidant Compounds. Antioxidants.

[103] Teerasong S., Jinnarak A., Chaneam S., Wilairat P., Nacapricha D. (2017). Poly(vinyl alcohol) capped silver nanoparticles for antioxidant assay based on seed-mediated nanoparticle growth. Talanta.

[104] Vernekar A.A., Sinha D., Srivastava S., Paramasivam P.U., D’Silva P., Mugesh G. (2014). An antioxidant nanozyme that uncovers the cytoprotective potential of vanadia nanowires. Nat. Commun..

[105] Medhe S., Bansal P., Srivastava M.M. (2014). Enhanced antioxidant activity of gold nanoparticle embedded 3,6-dihydroxyflavone: A combinational study. Appl. Nanosci..

[106] Bhattacharya K., Gogoi B., Buragohain A.K., Deb P. (2014). Fe_2_O_3_/C nanocomposites having distinctive antioxidant activity and hemolysis prevention efficiency. Mater. Sci. Eng. C.

[107] Leaves L. (2014). Antioxidant Activity by DPPH Radical Scavenging Method of Ageratum conyzoides. Orient.

[108] Foti M.C. (2015). Use and Abuse of the DPPH• Radical. J. Agric. Food Chem..

[109] Akhtar M.S., Rafiullah M., Shehata W.A., Hossain A., Ali M. (2022). Comparative phytochemical, thin layer chromatographic profiling and antioxidant activity of extracts from some Indian herbal drugs. J. Bioresour. Bioprod..

[110] Chen Z., Bertin R., Froldi G. (2013). EC_50_ estimation of antioxidant activity in DPPH* assay using several statistical programs. Food Chem..

[111] Liu C., Ge S., Yang J., Xu Y., Zhao M., Xiong L., Sun Q. (2016). Adsorption mechanism of polyphenols onto starch nanoparticles and enhanced antioxidant activity under adverse conditions. J. Funct. Foods.

[112] Logan S.R. (1982). The Origin and Status of the Arrhenius Equation. J. Chem. Educ..

[113] Ganguly P., Breen A., Pillai S.C. (2018). Toxicity of Nanomaterials: Exposure, Pathways, Assessment, and Recent Advances. ACS Biomater. Sci. Eng..

[114] Fu P.P., Xia Q., Hwang H.M., Ray P.C., Yu H. (2014). Mechanisms of nanotoxicity: Generation of reactive oxygen species. J. Food Drug Anal..

[115] Zhang H., Dunphy D.R., Jiang X., Meng H., Sun B., Tarn D., Xue M., Wang X., Lin S., Ji Z. (2012). Processing Pathway Dependence of Amorphous Silica Nanoparticle Toxicity: Colloidal vs. Pyrolytic. J. Am. Chem. Soc..

[116] Yan L., Zhao F., Wang J., Zu Y., Gu Z., Zhao Y. (2019). A Safe-by-Design Strategy towards Safer Nanomaterials in Nanomedicines. Adv. Mater..

[117] Nath D., Banerjee P. (2013). Green Nanotechnology–A New Hope for Medical Biology.

[118] Quester K., Avalos-Borja M., Castro-Longoria E. (2013). Biosynthesis and microscopic study of metallic nanoparticles. Micron.

[119] Sriranjani R., Srinithya B., Vellingiri V., Brindha P., Anthony S.P., Sivasubramanian A., Muthuraman M.S. (2016). Silver nanoparticle synthesis using Clerodendrum phlomidis leaf extract and preliminary investigation of its antioxidant and anticancer activities. J. Mol. Liq..

[120] Nichita C., Stamatin I. (2013). The antioxidant activity of the biohybrides based on carboxylated/hydroxylated carbon nanotubes-flavonoid compounds. Dig. J. Nanomater. Biostruct..

[121] Das D., Bhattacharyya S., Bhattacharyya M., Mandal P. (2022). Green chemistry inspired formation of bioactive stable colloidal nanosilver and its wide-spectrum functionalised properties for sustainable industrial escalation. Results Chem..

[122] Nagaich U., Gulati N., Chauhan S. (2016). Antioxidant and Antibacterial Potential of Silver Nanoparticles: Biogenic Synthesis Utilizing Apple Extract. J. Pharm..

[123] Fafal T., Taştan P., Tüzün B.S., Ozyazici M., Kivcak B. (2017). Synthesis, characterization and studies on antioxidant activity of silver nanoparticles using Asphodelus aestivus Brot. aerial part extract. S. Afr. J. Bot..

[124] Sudha A., Jeyakanthan J., Srinivasan P. (2017). Green synthesis of silver nanoparticles using Lippia nodiflora aerial extract and evaluation of their antioxidant, antibacterial and cytotoxic effects. Resour. Technol..

[125] AlSalhi M.S., Elangovan K., Ranjitsingh A.J.A., Murali P., Devanesan S. (2019). Synthesis of silver nanoparticles using plant derived 4-N-methyl benzoic acid and evaluation of antimicrobial, antioxidant and antitumor activity. Saudi J. Biol. Sci..

[126] Govindappa M., Hemashekhar B., Arthikala M.K., Ravishankar Rai V., Ramachandra Y.L. (2018). Characterization, antibacterial, antioxidant, antidiabetic, anti-inflammatory and antityrosinase activity of green synthesized silver nanoparticles using Calophyllum tomentosum leaves extract. Results Phys..

[127] Das D., Ghosh R., Mandal P. (2019). Biogenic synthesis of silver nanoparticles using S1 genotype of Morus alba leaf extract: Characterization, antimicrobial and antioxidant potential assessment. SN Appl. Sci..

[128] Oueslati M.H., Tahar L.B., Harrath A.H. (2020). Catalytic, antioxidant and anticancer activities of gold nanoparticles synthesized by kaempferol glucoside from Lotus leguminosae. Arab. J. Chem..

[129] Priya Velammal S., Devi T.A., Amaladhas T.P. (2016). Antioxidant, antimicrobial and cytotoxic activities of silver and gold nanoparticles synthesized using Plumbago zeylanica bark. J. Nanostructure Chem..

[130] Selvi A.M., Palanisamy S., Jeyanthi S., Vinosha M., Mohandoss S., Tabarsa M., You S.G., Kannapiran E., Prabhu N.M. (2020). Synthesis of Tragia involucrata mediated platinum nanoparticles for comprehensive therapeutic applications: Antioxidant, antibacterial and mitochondria-associated apoptosis in HeLa cells. Process Biochem..

[131] Zangeneh M.M., Ghaneialvar H., Akbaribazm M., Ghanimatdan M., Abbasi N., Goorani S., Pirabbasi E., Zangeneh A. (2019). Novel synthesis of Falcaria vulgaris leaf extract conjugated copper nanoparticles with potent cytotoxicity, antioxidant, antifungal, antibacterial, and cutaneous wound healing activities under in vitro and in vivo condition. J. Photochem. Photobiol. B Biol..

[132] Venugopalan R., Pitchai S., Devarayan K., Swaminathan V.C. (2020). Biogenic synthesis of copper nanoparticles using Borreria hispida (Linn.) extract and its antioxidant activity. Mater. Today Proc..

[133] Merugu R., Gothalwal R., Kaushik Deshpande P., De Mandal S., Padala G., Latha Chitturi K. (2021). Synthesis of Ag/Cu and Cu/Zn bimetallic nanoparticles using toddy palm: Investigations of their antitumor, antioxidant and antibacterial activities. Mater. Today Proc..

[134] Unuofin J.O., Oladipo A.O., Msagati T.A.M., Lebelo S.L., Meddows-Taylor S., More G.K. (2020). Novel silver-platinum bimetallic nanoalloy synthesized from Vernonia mespilifolia extract: Antioxidant, antimicrobial, and cytotoxic activities. Arab. J. Chem..

[135] Sharma C., Ansari S., Ansari M.S., Satsangee S.P., Srivastava M.M. (2020). Single-step green route synthesis of Au/Ag bimetallic nanoparticles using clove buds extract: Enhancement in antioxidant bio-efficacy and catalytic activity. Mater. Sci. Eng. C.

[136] Velsankar K., Sudhahar S., Maheshwaran G. (2019). Effect of biosynthesis of ZnO nanoparticles via Cucurbita seed extract on Culex tritaeniorhynchus mosquito larvae with its biological applications. J. Photochem. Photobiol. B Biol..

[137] Sharmila G., Muthukumaran C., Sangeetha E., Saraswathi H., Soundarya S., Kumar N.M. (2019). Green fabrication, characterization of Pisonia alba leaf extract derived MgO nanoparticles and its biological applications. Nano-Struct. Nano-Objects.

[138] Sharmila G., Thirumarimurugan M., Muthukumaran C. (2019). Green synthesis of ZnO nanoparticles using Tecoma castanifolia leaf extract: Characterization and evaluation of its antioxidant, bactericidal and anticancer activities. Microchem. J..

[139] Loganathan S., Shivakumar M.S., Karthi S., Nathan S.S., Selvam K. (2021). Metal oxide nanoparticle synthesis (ZnO-NPs) of Knoxia sumatrensis (Retz.) DC. Aqueous leaf extract and It’s evaluation of their antioxidant, anti-proliferative and larvicidal activities. Toxicol. Reports.

[140] Thakar M.A., Saurabh Jha S., Phasinam K., Manne R., Qureshi Y., Hari Babu V.V. (2021). X ray diffraction (XRD) analysis and evaluation of antioxidant activity of copper oxide nanoparticles synthesized from leaf extract of Cissus vitiginea. Mater. Today Proc..

[141] Akinola P.O., Lateef A., Asafa T.B., Beukes L.S., Hakeem A.S., Irshad H.M. (2020). Multifunctional titanium dioxide nanoparticles biofabricated via phytosynthetic route using extracts of Cola nitida: Antimicrobial, dye degradation, antioxidant and anticoagulant activities. Heliyon.

[142] Saravanakumar K., Sathiyaseelan A., Mariadoss A.V.A., Wang M.H. (2021). Antioxidant and antidiabetic properties of biocompatible ceria oxide (CeO_2_) nanoparticles in mouse fibroblast NIH3T3 and insulin resistant HepG2 cells. Ceram. Int..

[143] Dutta D., Mukherjee R., Patra M., Banik M., Dasgupta R., Mukherjee M., Basu T. (2016). Green synthesized cerium oxide nanoparticle: A prospective drug against oxidative harm. Colloids Surf. B Biointerfaces.

[144] Olvera Salazar A., García Hernández M., López Camacho P.Y., López Marure A., Reyes de la Torre A.I., Morales Ramírez Á., De J., Hernández Santiago F., Aguilera Vázquez L. (2016). Influence of Eu^3 +^ doping content on antioxidant properties of Lu_2_O_3_ sol-gel derived nanoparticles. Mater. Sci. Eng. C.

[145] Li Y., Fu R., Duan Z., Zhu C., Fan D. (2022). Artificial Nonenzymatic Antioxidant MXene Nanosheet-Anchored Injectable Hydrogel as a Mild Photothermal-Controlled Oxygen Release Platform for Diabetic Wound Healing. ACS Nano.

[146] Javed R., Usman M., Tabassum S., Zia M. (2016). Effect of capping agents: Structural, optical and biological properties of ZnO nanoparticles. Appl. Surf. Sci..

[147] Javed R., Ahmed M., Haq I., Nisa S., Zia M. (2017). PVP and PEG doped CuO nanoparticles are more biologically active: Antibacterial, antioxidant, antidiabetic and cytotoxic perspective. Mater. Sci. Eng. C.

[148] Tsai Y.H., Yang Y.N., Ho Y.C., Tsai M.L., Mi F.L. (2018). Drug release and antioxidant/antibacterial activities of silymarin-zein nanoparticle/bacterial cellulose nanofiber composite films. Carbohydr. Polym..

[149] Kim S.J., Chung B.H. (2016). Antioxidant activity of levan coated cerium oxide nanoparticles. Carbohydr. Polym..

[150] Gao L., Zhuang J., Nie L., Zhang J., Zhang Y., Gu N., Wang T., Feng J., Yang D., Perrett S. (2007). Intrinsic peroxidase-like activity of ferromagnetic nanoparticles. Nat. Nanotechnol..

[151] Alvarez Echazú M.I., Olivetti C.E., Peralta I., Alonso M.R., Anesini C., Perez C.J., Alvarez G.S., Desimone M.F. (2018). Development of pH-responsive biopolymer-silica composites loaded with Larrea divaricata Cav. extract with antioxidant activity. Colloids Surf. B Biointerfaces.

[152] Lee R.J., Tamm T., Temmer R., Aabloo A., Kiefer R. (2013). Two formation mechanisms and renewable antioxidant properties of suspensible chitosan-PPy and chitosan-PPy-BTDA composites. Synth. Met..

[153] Liu X., Li D., Liang Y., Lin Y., Liu Z., Niu H., Xu Y. (2021). Establishment of anti-oxidation platform based on few-layer molybdenum disulfide nanosheet-coated titanium dioxide nanobelt nanocomposite. J. Colloid Interface Sci..

[154] Sachdev A., Gopinath P. (2016). Monitoring the Intracellular Distribution and ROS Scavenging Potential of Carbon Dot–Cerium Oxide Nanocomposites in Fibroblast Cells. ChemNanoMat.

[155] Rajeswari R., Gurumallesh Prabu H. (2020). Palladium–Decorated reduced graphene oxide/zinc oxide nanocomposite for enhanced antimicrobial, antioxidant and cytotoxicity activities. Process Biochem..

[156] Fu Y., Zhang J., Wang Y., Li J., Bao J., Xu X., Zhang C., Li Y., Wu H., Gu Z. (2021). Reduced polydopamine nanoparticles incorporated oxidized dextran/chitosan hybrid hydrogels with enhanced antioxidative and antibacterial properties for accelerated wound healing. Carbohydr. Polym..

[157] Battaglini M., Marino A., Carmignani A., Tapeinos C., Cauda V., Ancona A., Garino N., Vighetto V., La Rosa G., Sinibaldi E. (2020). Polydopamine Nanoparticles as an Organic and Biodegradable Multitasking Tool for Neuroprotection and Remote Neuronal Stimulation. ACS Appl. Mater. Interfaces.

[158] Muniyappan N., Pandeeswaran M., Amalraj A. (2021). Green synthesis of gold nanoparticles using Curcuma pseudomontana isolated curcumin: Its characterization, antimicrobial, antioxidant and anti- inflammatory activities. Environ. Chem. Ecotoxicol..

[159] Huang J., Wang Q., Li T., Xia N., Xia Q. (2017). Nanostructured lipid carrier (NLC) as a strategy for encapsulation of quercetin and linseed oil: Preparation and in vitro characterization studies. J. Food Eng..

[160] Karimi N., Ghanbarzadeh B., Hamishehkar H., Mehramuz B., Kafil H.S. (2018). Antioxidant, Antimicrobial and Physicochemical Properties of Turmeric Extract-Loaded Nanostructured Lipid Carrier (NLC). Colloids Interface Sci. Commun..

[161] Huang X., Huang X., Gong Y., Xiao H., McClements D.J., Hu K. (2016). Enhancement of curcumin water dispersibility and antioxidant activity using core-shell protein-polysaccharide nanoparticles. Food Res. Int..

[162] Zhang X., Guo X., Kang X., Yang H., Guo W., Guan L., Wu H., Du L. (2020). Surface functionalization of pegylated gold nanoparticles with antioxidants suppresses nanoparticle-induced oxidative stress and neurotoxicity. Chem. Res. Toxicol..

[163] Massaro M., Riela S., Guernelli S., Parisi F., Lazzara G., Baschieri A., Valgimigli L., Amorati R. (2016). A synergic nanoantioxidant based on covalently modified halloysite–trolox nanotubes with intra-lumen loaded quercetin. J. Mater. Chem. B.

[164] Oliveira A.I., Pinho C., Fonte P., Sarmento B., Dias A.C.P. (2018). Development, characterization, antioxidant and hepatoprotective properties of poly(Ɛ-caprolactone) nanoparticles loaded with a neuroprotective fraction of Hypericum perforatum. Int. J. Biol. Macromol..

[165] Lee G.H., Lee S.J., Jeong S.W., Kim H.C., Park G.Y., Lee S.G., Choi J.H. (2016). Antioxidative and antiinflammatory activities of quercetin-loaded silica nanoparticles. Colloids Surf. B Biointerfaces.

[166] Sotiriou G.A., Blattmann C.O., Deligiannakis Y. (2016). Nanoantioxidant-driven plasmon enhanced proton-coupled electron transfer. Nanoscale.

[167] De Cristo Soares Alves A., Mainardes R.M., Khalil N.M. (2016). Nanoencapsulation of gallic acid and evaluation of its cytotoxicity and antioxidant activity. Mater. Sci. Eng. C.

[168] Lee J., Choi K.H., Min J., Kim H.J., Jee J.P., Park B.J. (2017). Functionalized ZnO nanoparticles with gallic acid for antioxidant and antibacterial activity against methicillin-resistant S. aureus. Nanomaterials.

[169] Bumbudsanpharoke N., Choi J., Park I., Ko S. (2015). Facile Biosynthesis and Antioxidant Property of Nanogold-Cellulose Fiber Composite. J. Nanomater..

[170] Marulasiddeshwara M.B., Dakshayani S.S., Sharath Kumar M.N., Chethana R., Raghavendra Kumar P., Devaraja S. (2017). Facile-one pot-green synthesis, antibacterial, antifungal, antioxidant and antiplatelet activities of lignin capped silver nanoparticles: A promising therapeutic agent. Mater. Sci. Eng. C.

[171] Baschieri A., Amorati R., Benelli T., Mazzocchetti L., D’angelo E., Valgimigli L. (2019). Enhanced antioxidant activity under biomimetic settings of ascorbic acid included in halloysite nanotubes. Antioxidants.

[172] Arriagada F., Günther G., Morales J. (2020). Nanoantioxidant–based silica particles as flavonoid carrier for drug delivery applications. Pharmaceutics.

[173] Du L., Suo S., Wang G., Jia H., Liu K.J., Zhao B., Liu Y. (2013). Mechanism and cellular kinetic studies of the enhancement of antioxidant activity by using surface-functionalized gold nanoparticles. Chem. A Eur. J..

[174] Huang Y., Liu Z., Liu C., Ju E., Zhang Y., Ren J., Qu X. (2016). Self-Assembly of Multi-nanozymes to Mimic an Intracellular Antioxidant Defense System. Angew. Chemie.

[175] Hemmati S., Zangeneh M.M., Zangeneh A. (2020). CuCl2 anchored on polydopamine coated-magnetic nanoparticles (Fe_3_O_4_@PDA/Cu(II)): Preparation, characterization and evaluation of its cytotoxicity, antioxidant, antibacterial, and antifungal properties. Polyhedron.

